# OsCCRL1 is Essential for Phenylpropanoid Metabolism in Rice Anthers

**DOI:** 10.1186/s12284-023-00628-1

**Published:** 2023-02-27

**Authors:** Lisha Zhang, Lintao Zheng, Jingwen Wu, Yang Liu, Weichi Liu, Guanghua He, Nan Wang

**Affiliations:** Key Laboratory of Application and Safety Control of Genetically Modified Crops, College of Agronomy and Biotechnology, Rice Research Institute, Southwest University, Chongqing, 400715 China

**Keywords:** Male sterility, Phenylpropanoid metabolism, Tapetum degradation, OsMYB103/OsMYB80/OsMS188/BM1

## Abstract

Phenylpropanoid metabolism and timely tapetal degradation are essential for anther and pollen development, but the underlying mechanisms are unclear. In the current study, to investigate this, we identified and analyzed the male-sterile mutant, *osccrl1* (*cinnamoyl coA reductase-like 1*), which exhibited delayed tapetal programmed cell death (PCD) and defective mature pollen. Map-based cloning, genetic complementation, and gene knockout revealed that *OsCCRL1* corresponds to the gene *LOC_Os09g32020*.2, a member of SDR (short-chain dehydrogenase/reductase) family enzyme. OsCCRL1 was preferentially expressed in the tapetal cells and microspores, and localized to the nucleus and cytoplasm in both rice protoplasts and *Nicotiana benthamiana* leaves. The *osccrl1* mutant exhibited reduced CCRs enzyme activity, less lignin accumulation, delayed tapetum degradation, and disrupted phenylpropanoid metabolism. Furthermore, an R2R3 MYB transcription factor OsMYB103/OsMYB80/OsMS188/BM1, involved in tapetum and pollen development, regulates the expression of *OsCCRL1*. Finally, the *osmyb103 osccrl1* double mutants, exhibited the same phenotype as the *osmyb103* single mutant, further indicating that OsMYB103/OsMYB80/OsMS188/BM1 functions upstream of OsCCRL1. These findings help to clarify the role of phenylpropanoid metabolism in male sterility and the regulatory network underlying the tapetum degradation.

**Supplementary Information:**

The online version contains supplementary material available at 10.1186/s12284-023-00628-1.

## Background

The phenylpropanoid metabolism produces many important secondary metabolites, such as lignin, flavonoids, lignans, phenylpropanoid esters, hydroxycinnamic acid amides, coumarins, and sporopollenin, which are vital for plant development and survival (Knaggs 2003; Dong and Lin 2021). This pathway begins with three general enzymes: phenylalanine ammonialyase (PAL), cinnamic acid 4-hydroxylase (C4H), and 4-coumarate-CoA ligase (4CL) (Fraser and Chapple 2011). Subsequently, it splits into different branches to synthesize different products. Increasing numbers of studies have shown that phenylpropanoid metabolism is closely related to fertility in plants.

Sporopollenin, the main constituent of pollen exine, is a highly resistant biopolymer composed of many substances, such as aromatics, phenolics, long-chain aliphatic acids, and some phenylpropane metabolites, like ferulate, *p*-hydroxybenzoate, *p*-coumarate and lignin units (Domínguez et al. 1999; Bubert et al. 2002; Ahlers et al. 2003), and interfering with the phenylpropanoid pathway could affect the biosynthesis of sporopollenin (Xue et al. 2020). Besides, anther cuticle is also important to protect pollen from external environment, and it is mainly composed of cutin and wax. The generation of cutin and wax also requires the phenylpropane metabolites such as triterpenoids, flavonoids, phenylpropanoids esters. Therefore, when the anther cuticle was destroyed, a whitish anther epidermis often could result (Jetter et al. 2006).

Lignin, an important end-product in phenylpropanoid pathway, is also indispensable for anther development. The lignification and thickening of anther endothecium layer mediate anther dehiscence, thereby affecting fertility (Zhang and Wilson 2009; Gui et al. 2011). Suppressing *Os4CL3* (*4-Coumarate: coenzyme A Ligase 3*) expression causes a wrinkled exterior appearing on the anther surface and the failure of the endothecium layer lignification, resulting in anther dehiscences and ultimately infertility (Schilmiller et al. 2009; Gui et al. 2011). Cinnamoyl CoA reductase (CCR) and cinnamyl alcohol dehydrogenase (CAD), two important enzymes in the biosynthesis of lignin units, also play important roles in plant fertility (Baucher et al. 1995; Lacombe et al. 1997; Barros et al. 2019). In *Arabidopsis*, the simultaneous repression of CCR and CAD severely damages the lignin structure and reduces the lignin content. The *Arabidopsis cad c cad d ccr1* triple mutant displays a severe dwarf phenotype and male sterility (Thévenin et al. 2011). Among them, CCR is the first committed enzyme in the monolignol pathway for lignin biosynthesis and catalyzes the conversion of hydroxycinnamoyl-CoAs to hydroxycinnamaldehydes. And it is accepted that CCRs involved in lignification using feruloyl-CoA as substrates, mainly in grasses (Ma et al. 2007; Escamilla-Treviño et al. 2010). Besides, CCRs are considered members of SDR (short-chain dehydrogenase/reductase) superfamily, whose *N*-terminal region binds the coenzymes NAD(P)(H) and the *C*-terminal region constitutes the substrate binding part. SDR family is a large family that contains more than 3000 members, with a substrate spectrum ranging from alcohols, sugars, steroids and aromatic compounds to xenobiotics (Kallberg et al. 2002).

Flavonoid, another major phenylpropanoid metabolites, also takes part in male fertility. Except for sporopollenin, the pollen exine is also covered by flavonoid glycosides, and the biosynthesis of pollen intine also requires the flavonoid metabolites. Therefore, the flavonoid metabolites are necessary for the integrity of pollen exine and intine (Grunewald et al. 2020; Battat et al. 2019). Chalcone synthase (CHS) catalyzes the initiation of flavonoid biosynthesis and the rice *oschs1* mutant produces flavonoid-depleted pollen and shows a loss of male fertility (Wang et al. 2020).

In general, when the phenylpropanoid metabolic pathway in anthers is affected, processes such as the development of the anther epidermis, sporopollenin, and the pollen wall, as well as anther dehiscence, are abnormal. Therefore, phenylpropanoid metabolism is vital for anther and pollen development.

In this study, we isolated the male-sterilemutant *osccrl1* from *indica* rice and determined that it has abnormal anthers and defective mature pollen. *OsCCRL1* was localized to the nucleus and cytoplasm and expressed in the tapetum and microspores. The *osccrl1* mutant exhibited reduced CCRs enzyme activity, less lignin accumulation, disturbed phenylpropanoid metabolism, and delayed tapetum degradation. We also determined that the transcription factor OsMYB103/OsMYB80/OsMS188/BM1 regulates *OsCCRL1* expression. At the genetic level, the *osmyb103 osccrl1* double mutants showed the same phenotype as *osmyb103* single mutant, indicating that OsCCRL1 functions the down stream of OsMYB103/OsMYB80/OsMS188/BM1. These findings provide evidence for the importance of phenylpropanoid metabolism in rice male sterility and enrich the network underlying the tapetal PCD.

## Materials and Methods

### Plant Material

The rice (*Oryza sativa* L. *ssp. indica*) *osccrl1* mutant was derived from ‘Xinong 1B’ population treated by 1% (v/v) ethyl methanesulfonate (EMS) and inherited stably after more than three years. In phenotypic characterization and cytological analyse experiments, ‘Xinong 1B’ was used as the wild-type (WT) control. Male-sterile plants in the F_2_ population generated from the cross between ‘Jinhui 10’ and the *osccrl1* mutant were selected to *OsCCRL1*. All plants including transgenic plants were bred in Southwest University, Chongqing, China, under natural conditions.

### Microscopy

The phenotypes of the *osccrl1* mutant and the wild type were compared over the entire growth period under natural conditions. During the flowering period, wild type and *osccrl1* mutant spikelets and anthers were randomly selected and observed using a SMZ1500 stereomicroscope (Nikon, Tokyo, Japan). For scanning electron microscopy (SEM) observations, the anther epidermis and inner surface were observed using a SU3500 scanning electron microscope (Hitachi, Tokyo, Japan) under an accelerating voltage of 5.0 kV at − 20 °C. To analyze pollen fertility, mature pollen grains were stained with 1% (w/v) I_2_/KI solution and photographed using an Eclipse E600 microscope (Nikon). To observe anther development in the wild type and *osccrl1* mutant, anthers at different stages were collected and embedded for semi-section as described previously (Li et al. 2006). Briefly, anthers were pre-fixed in 2.5% (v/v) glutaraldehyde and 2% (w/v) paraformaldehyde at 4 °C, and then post-fixed with 1% (w/v) osmium tetroxide at room temperature. Subsequently, samples were dehydrated with a graded ethanol series [50% (v/v), 70% (v/v), 85% (v/v), 95% (v/v), 100% (v/v), 100% (v/v), and 100% (v/v)] and embedded in SPI-PON 812 resin (Shanghai Physion Instruments, Shanghai, China). 2 μm sections were prepared using an EM-UC6 microtome (Leica, Wetzal, Germany), and then stained with 0.5% (w/v) toluidine blue. For TUNEL analyses of the wild type and *osccrl1* mutant anther, anthers at different stages were collected and embedded in paraffin (Sigma-Aldrich, St Louis, MO, USA) in accordance with a previously described method (Fu et al. 2014). In situ analyses of nick-end labeled nuclear DNA fragments in wild-type and *osccrl1* anthers was performed with the DeadEnd™ Fluorometric TUNEL Kit (Promega, Madison, WI, USA) in accordance with the manufacturer’s recommendations. TUNEL signals were analyzed using a LSM 800 confocal laser scanning microscopy (Zeiss, Jena, Germany). The pretreatment and embedding of wild-type and *osccrl1* anthers for transmission electron microscopy (TEM) analyse were similar to thatin semi-thin section. Using an EM-UC6 microtome, 60 nm ultrathin sections were cut, and then stained with uranyl acetate and citrate aqueous. After staining, the sections were observed and photographed using H-7500 TEM microscope (Hitachi).

### Genetic Analysis and Fine-Mapping of *OsCCRL1*

The *osccrl1* mutant was crossed with ‘Jinhui 10’ to generate the F_1_ population and the F_2_ population was derived by the self-fertilization of F_1_ individuals. The F_2_ individuals that exhibited male sterility were selected to map *OsCCRL1* gene. Gene mapping was conducted using simple sequence repeat markers obtained from the publicly available rice databases Gramene (http://www.gramene.org) and the Rice Genomic Research Program (http://rgp.dna.affrc.go.jp/E/publicdata/caps/index.html). Insertionand deletion markers were developed from comparison of genomic sequences for ‘Xinong 1B’ and ‘Jinhui 10’.

### Complementary Verification of *OsCCRL1*

To complement the male-sterile phenotype of the *osccrl1* mutant, a 6383 bp genomic fragment from the wild-type genomic DNA was amplified and cloned into the binary vector pCAMBIA1301. The complementary vector was introduced into the *osccrl1* mutant using the *Agrobacterium tumefaciens*-mediated method (Ma et al. 2017). The primer sequences used for vector construction are listed in Additional file 1: Table S1.

### CRISPR-CAS9 Mediated Gene Editing

In regularly interspaced short palindromic repeats (CRISPR)-CAS9 (CRISPR-associated protein 9) mediated gene editing, the specific and low mop rate target sequences were designed according ‘CRISPR direct’ and ‘RNA-fold’ web server. The designed knockout site was constructed into the knockout vector pCAR-CAS9 according to the system (Ma and Liu 2016). The vectors were introduced into the corresponding plants using *Agrobacterium tumefaciens*-mediated method. The primer sequences used for vector construction are listed in Additional file 1: Table S1.

### Reverse Transcription Real-Time Quantitative PCR and In Situ Hybridization

Total RNAs from isolated tissue were extracted and purified using the RNAprep Pure Plant Kit (Tiangen, Beijing, China). 1 µg RNA was used to synthesis cDNA with the SuperScript® III Reverse Transcriptase Kit (Invitrogen, Shanghai, China). After reverse transcription, all samples were diluted with sterilized water. The reverse transcription real-time quantitative PCR (RT-qPCR) analysis was performed on the ABI 7500 Sequence Detection System (Thermo Fisher Scientific, Waltham, MA, USA) using the SYBR® Premix Ex Taq™ GC Kit (Takara, Dalian, China). Using *OsACTIN1* as an endogenous control, three replicates were performed and the normalization of relative expression was calculated using the ∆∆*C*_t_ method (Livak et al. 2001). For mRNA in situ hybridization of OsCCRL1, the 331 bp specific probe of *OsCCRL1* was amplified and labeled using the DIG RNA Labeling Kit (Roche, Basel, Switzerland). Pretreatment of sections, hybridization, and immunological detection were performed as described previously (Ma et al. 2017). The primer sequences used for RT-qPCR and in situ hybridization are listed in Additional file 1: Table S1.

### Subcellular Localization

To generate fusion proteins with GFP (green fluorescent protein), the full coding region of *OsCCRL1* and *LOC_Os09g32020.1* without stop codon was amplified and cloned into pAN580 (2 × *35S*-GFP-Nos) vector. The generated plasmids, 2 × *35S*::OsCCRL1-GFP and 2 × *35S*::LOC_Os09g32020.1-GFP, were introduced into rice protoplasts following a previously described method (Ma et al. 2017). The nuclear-localized marker OsH2B-mCherry and endoplasmic reticulum (ER)-localized marker OsHDEL-mCherry were used. After incubation at 28 °C overnight, fluorescence signals were observed using a LSM 800 confocal laser scanning microscope (Zeiss). For the subcellular localization of *Arabidopsis* protein, the full-length coding sequence of *AtTKPR1* and *AtTKPR2* was also cloned into the pAN580 vector and introduced into *Arabidopsis* protoplasts as a previously described method (Wang et al. 2015). For the subcellular localization in *Nicotiana benthamiana*, the full-length coding sequence of *OsCCRL1*, *AtTKPR1* and *AtTKPR2* was cloned into the pCAMBIA1300 (*35S*-GFP-Nos) vector driven by one CaMV *35S* promoter. Constructs were introduced into *Nicotiana benthamiana* leaf cells by *Agrobacterium-*mediated infiltration. The nuclear was indicated by 4',6-diamidino-2-phenylindole (DAPI). The primer sequences used for vector construction are listed in Additional file 1: Table S1.

### Western-Blot Analysis of Extracted Nuclear and Cytoplasmic Protein

The cytoplasmic and nuclear proteins from tobacco leaf cells expressing *35S*::OsCCRL1-GFP was extracted with previous methods and the western-blot detection was performed (Pandey et al. 2006; Xu et al. 2006; Chen et al. 2014). Histone antibodies was used as the internal reference antibody for detecting nuclear protein. Tubulin antibodies was used as the internal reference antibody for detecting cytoplasmic protein.

### Phylogenetic Analysis

To identify the putative homologs of OsCCRL1, its protein sequence was used as a BLAST query on the ‘Gramene’ and ‘NCBI’ database. Phylogenetic tree construction was conducted by Maximum Likelihood method. The protein sequences used for phylogenetic tree are listed in Additional file 1: Table S2.

### Total CCRs Enzyme Activity, Lignin Staining and Content of Anther

Under the protection of liquid nitrogen, total CCRs enzyme solutions were extracted from equal amounts anthers in WT and *osccrl1* mutant according to the previous method, and were measured the absorbance of A366 by adding the feruloyl-coA as the substrate (Goffner et al. 1994). Briefly, The reaction mixture consisted of 0.1 mM NADPH, 70 μM feruloyl-coA, and different volumes of extracted CCRs from WT and *osccrl1* mutant in 100 mM sodium/potassium phosphate buffer (pH 6.25) in a total volume of 500 μL. For lignin staining, the anthers of stage 12 were dissected and stained with phloroglucinol-HCl (Hao et al. 2014), then photographed using an Eclipse E600 microscope (Nikon). To quantify the lignin content, the extraction and quantification from wild-type and *osccrl1* mutant spikelet using a previous method with three biological replicates (Moreira-Vilar et al. 2014; Borah and Khurana 2018). Briefly, 0.2 mg sample was rinsed in H-buffer (50 mM Tris–HCL, 10 g/L Triton, 1 M NaCl, PH 8.3) and acetone several times. Then grind them into powder under the protection of liquid nitrogen after drying. All samples were placed into a screw-cap centrifuge tube containing the reaction mixture (1.2 mL of thioglycolic acid and 6 mL of 2 M HCl) and heated (98 °C, 4 h). A standard curve for lignin (alkali, Aldrich) by measuring the absorbance of A280 was generated. The lignin content in wild-type and *osccrl1* mutant spikelet was calculated by referring to the standard curve.

### The Enzyme Activity of OsCCRL1 In Vitro

The full-length coding region of OsCCRL1 and osccrl1 was amplified and recombined into the pGEX-4 T-1 vector, and then transformed into *Escherichia coli* BL21 (DE3) to obtain the OsCCRL1-GST and osccrl1-GST fusion protein. The CCR enzyme activity of OsCCRL1 and osccrl1 was measured according to the method (Lüderitz and Grisebach 1981). The reaction mixture consisted of 0.1 mM NADPH, 100 μM feruloyl-coA, and 5 μg of purified recombinant protein in 100 mM sodium/potassium phosphate buffer (pH 6.25) in a total volume of 500 μL. The enzyme reactions were carried out at 30 °C and the absorbance of A366 was monitored. The primer sequences used for vector construction are listed in Additional file 1: Table S1.

### Transient Expression Assay

The promoter of *OsCCRL1* (1576 bp) were amplified and cloned into the pGreenII0800-LUC double-reporter vector (firefly luciferase and renilla luciferase). The full-length coding region of OsMYB103 was amplified and cloned to pAN580-no-GFP (2 × *35S*-Nos) vector. The constructs were then transformed into rice protoplasts. After overnight incubation at 28 °C, LUC and Renilla (REN) luciferase activities were measured using the Dual Luciferase Assay Kit (Promega, USA), and analysed using the Luminoskan Ascent Microplate Luminometer (Thermo Fisher Scientific, USA) according to the manufacturer’s instructions. The pAN580-no-GFP was used as an control. At least six transient measurements were conducted for each assay. The primer sequences used for vector construction are listed in Additional file 1: Table S1.

### Chromatin Immunoprecipitation Analysis

The ChIP assays were performed in *Ubi::OsMYB103-GFP* transgenic anthers using the EpiQuik™ Plant ChIP kit (EpiGentek, Farmingdale, NY, USA) in accordance with the manufacturer’s recommendations. Briefly, fresh tissues were crosslinked with 1% (v/v) formaldehyde solution under vacuum and decrosslinked by adding 2 M glycine solution (final concentration 0.125 M). The protein–DNA complexes were sheared into 200–500 bp fragments by sonication from the protease inhibitor cocktail protection. After sonication, a portion of chromatin was reverse-crosslinked and used as the input DNA control for ChIP-qPCR analysis. A dilution of the resulting chromatin was incubated with anti-GFP antibody ab290 (Abcam, Cambridge, UK) or anti-IgG antibody at room temperature with orbital shaking. The amount of immunoprecipitated genomic DNA was performed on an ABI 7500 Sequence Detection System (Thermo Fisher) with three biological repetitions. The calculation of relative enrichment followed a previously described method (Li et al. 2011). Relative enrichment was measured by comparing the input and ChIP groups. Normal anti-IgG antibody was used as a negative control. The primer sequences used for vector construction and ChIP-qPCR analysis are listed in Additional file 1: Table S1.

### Electrophoretic Mobility Shift Assay

The full coding sequence of OsMYB103 was cloned into the pET-28a vector (Novagen) and then transformed into *Escherichia coli* strain BL21(DE3) to obtain the OsMYB103-HIS fusion protein. The electrophoretic mobility shift assay (EMSA) was performed using the LightShift™ Chemiluminescent EMSA Kit (BersinBio, Guangzhou, China) in accordance with the manufacturer’s instructions. The enriched regulatory sequences ‘P2’ and ‘P7’ in ChIP-qPCR analysis were synthesized to shorter sequences and annealed to biotin-labeled double-stranded DNA probe (Novagen), named ‘P2s’ and ‘P7s’, respectively. Binding reactions were then separated in 6% acrylamide negative gel and transferred to nylon membranes. Through blocking and washing, membranes were placed in a film cassette and exposed to X-ray film for 5 min. The primer sequences used for vector construction and DNA probes are listed in Additional file 1: Table S1.

### The Callose Assays

For callose assays, anthers at stage 8b were collected, dehydrated, and embedded in paraffin (Sigma, St Louis, MO, USA). 10 μm thickness paraffin sections were cut and stained with 0.25% (w/v) aniline blue (Sigma-Aldrich) following a previous method (Wan et al. 2011). The stained sections were observed with a Nikon-80 fluorescence microscope (Nikon) under ultraviolet light condition.

### Sudan Red 7B Staining

For lipidic staining of anther, anthers at the mature pollen stage were soaked in a solution containing 0.1% (w/v) sudan red 7B, 50% (v/v) polyethylene glycol-400, 45% (v/v) glycerol. The processing and staining were performed according to the method reported previously (Xu et al. 2017).

### Accession Numbers

Sequence data from this article can be found in the GenBank/EMBL libraries under the following accession numbers: *OsMYB103* (*LOC_Os04g39470*), *OsCCRL1* (*LOC_Os09g32020.2*), *AtTKPR1* (*AT4G35420*), *AtTKPR2* (*AT1G68540*), *Os4CL3* (*LOC_Os02g08100*), *OsCHS1* (*LOC_Os11g32650)*, *OsAP25* (*LOC_Os03g08790*), *OsAP37* (*LOC_Os04g37570*), *C6* (*LOC_Os11g37280*), *OsPKS2* (*LOC_Os07g22850*).

## Results

### Isolation and Phenotypic Characterization of the *osccrl1* Mutant

**Fig. 1:**
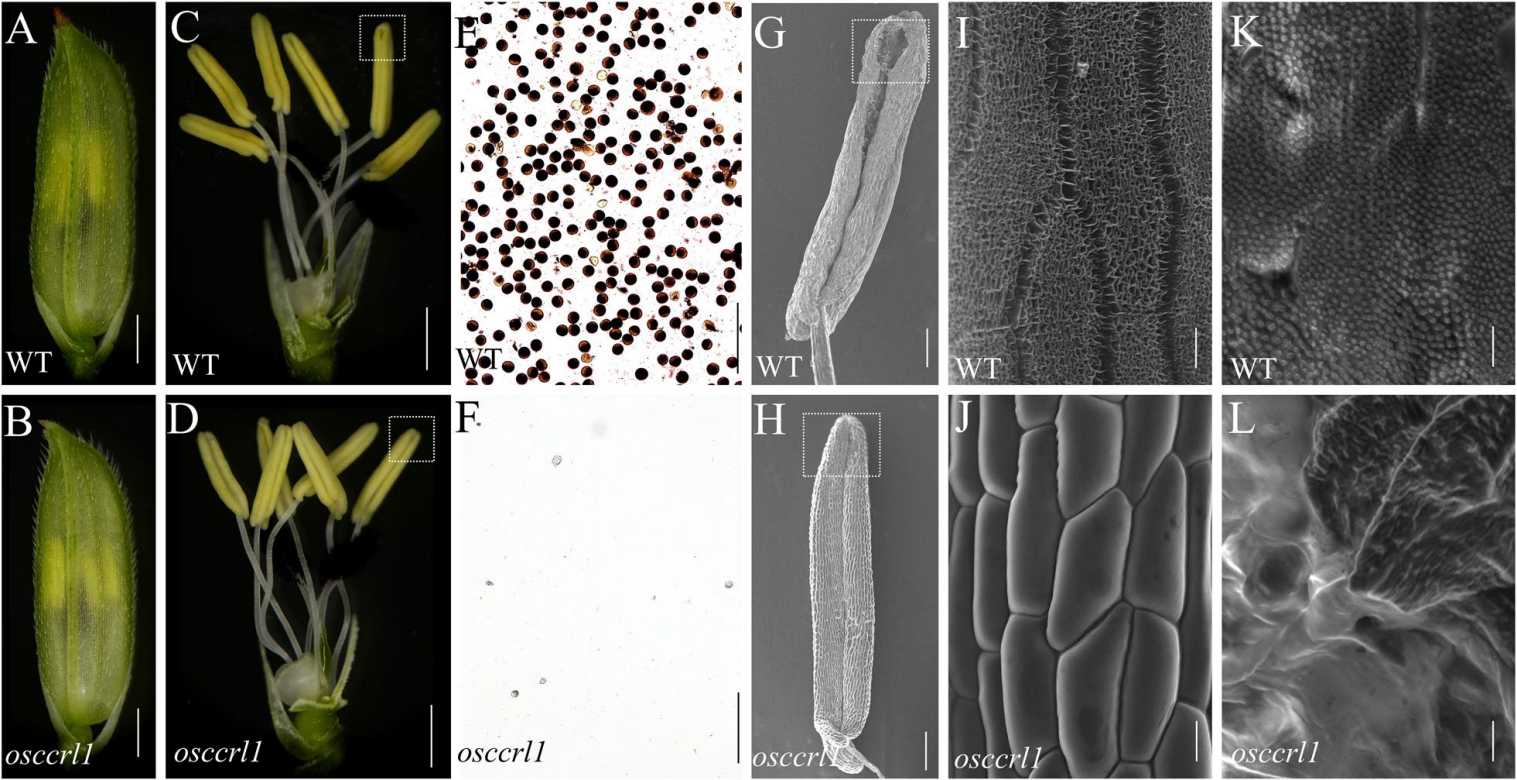
Phenotypic comparison between the WT and the *osccrl1* mutant. **A**, **B** Spikelet of the wild type and *osccrl1* mutant. Scale bars, 5 mm. **C**, **D** Spikelet of the wild type and *osccrl1* mutant after removal of the palea and lemma. Scale bars, 5 mm. **E**, **F** Pollen grains of the wild type and *osccrl1* mutant stained by I_2_/KI solution. Scale bars, 200 μm. **G**, **H** The scanning electron microscopy of anther in wild type and *osccrl1* mutant. Scale bars, 200 μm. **I**, **J** The scanning electron microscopy of anther epidermis surface in wild type and *osccrl1* mutant. Scale bars, 50 μm. **K**, **L** The scanning electron microscopy of anther inner surface in wild type and *osccrl1* mutant. Scale bars, 50 μm

To identify additional genes that function in anther and pollen development, we screened a rice mutant library generated by ethyl methanesulfonate (EMS) mutagenesis of *Oryza sativa* L. ssp. *indica* ‘Xinong 1B’. We selected one mutant with complete male sterility, *osccrl1* (*cinnamoyl coA reductase-like 1*), for further analysis. At the vegetative stages, there were no obvious phenotypic differences between the wild type and *osccrl1* mutant (data not shown). At the flowering stage, both wild-type and *osccrl1* spikelets developed normally, and their floral organs were visible (Fig. 1A–D). However, compared to the wild type, *osccrl1* mutant anthers dehisce abnormally, without any cracking of the apexes or bases (Fig. 1C, D). In addition, pollen iodine potassium iodide (I_2_/KI) staining revealed no viable pollen in *osccrl1* anther, which showed *osccrl1* was completely male-sterile (Fig. 1E, F).

We performed scanning electron microscopy of the anthers epidermis and inner surface to further investigate the morphological differences between the wild type and *osccrl1* mutant. The wild-type anthers clearly dehisced, whereas *osccrl1* mutant anthers did not dehisce normally (Fig. 1G, H). In addition, a well-ordered nano-ridged structure formed by anther cuticle appeared on the wild-type anthers epidermis, whereas the *osccrl1* anther epidermis at the same stage appeared smoother, with reduced deposition of the cutin and wax polymers (Fig. 1I, J). On the anther inner surface, many dense Ubisch bodies with sharp protrusions were uniformly distributed in the wild type, whereas almost no Ubisch bodies were observed in the *osccrl1* mutant (Fig. 1K, L).

**Fig. 2:**
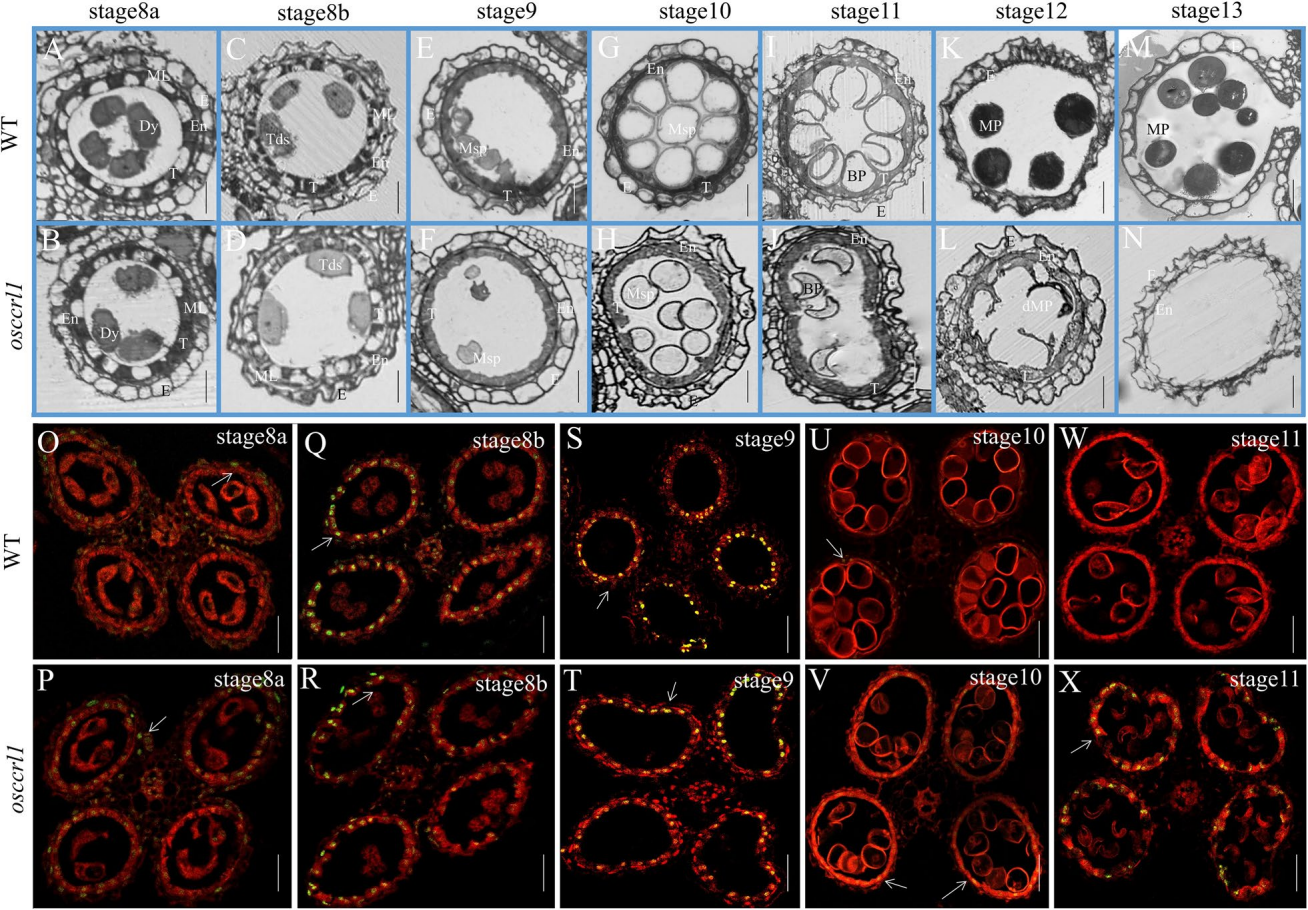
Semi-thin section and TUNEL assay of the wild type and *osccrl1* mutant. **A** − **N** Semi-thin sections of anthers at stages 8a to 13 in the wild type and *osccrl1* mutant. The images show a single locule of an anther. E, epidermis; En, endothecium; M, middle layer; T, tapetum; Dy, dyad cell; Tds, tetrads; Msp, microspore; BP, bicellular pollen; MP, mature pollen; dMP, mature pollen. Scale bars, 25 μm. **O** − **X** TUNEL analyses of wild-type and *osccrl1* anthers at stages 8a to 11. Propidium iodide staining is indicated by red fluorescence and TUNEL positive staining is indicated by yellow to green fluorescence (arrow indication). Scale bars, 100 μm

Semi-thin transverse sections were performed to further examine the anther morphological defects in wild type and *osccrl1* mutant. At the stages of 8a and 8b, both wild-type and *osccrl1* mutant anthers contained four layers: the epidermis, endothecium, middle layer, and tapetum. At the stage 8a, the tapetal cells appeared vacuolated, and the meiocytes formed ellipsoidal shaped dyads. At stage 8b, the tapetal cell continued the process of vacuolation, and the dyads had undergone meiosis to form a haploid tetrad (Fig. 2A–D). At stage 9, no obvious morphological difference was visible between wild-type and *osccrl1* mutant anthers, as they all contained three cellular layers and free globular microspores (Fig. 2E, F). The morphological difference between *osccrl1* mutant and wild-type anthers began to appear at stage 10. At this stage, the tapetum appeared thicker in *osccrl1* mutant than that in the wild type, and the *osccrl1* microspores showed smaller spherical and uneven distribution in anther chamber comparing with that in wild type (Fig. 2G, H). At stage 11, wild-type microspore had undergone mitotic division and appeared the falcate shape, and the tapetum had undergone normal PCD and become denser. In the *osccrl1* mutant, the microspores also appeared to have undergone mitosis and become sickle-shaped, but the tapetal cells showed a delay in PCD, as they contained a thicker tapetum layer than that in the wild type (Fig. 2I, J). At stage 12, wild-type microspores were darkly stained and were filled with starch. By contrast, *osccrl1* microspores were abnormally shrunken, showing an infertile phenotype (Fig. 2K, L). And at stage 13, the wild-type anther normally dehisced, whereas *osccrl1* mutant anthers did not dehisce and had none pollen (Fig. 2M, N).

The PCD of tapetal cells is characterized by the random cleavage of nuclear DNA, which can be detected through a terminal deoxynucleotidyl transferase-mediated dUTP nick-end labelling (TUNEL) assay. We therefore performed a TUNEL assay of wild-type and *osccrl1* anthers to test whether the PCD of tapetal cells was defective in the *osccrl1* mutant. At stage 8a, 8b, and 9, a positive TUNEL signal was observed in both wild-type and *osccrl1* tapetal cells (Fig. 2O–T), and the TUNEL fluorescence signal reached the maximum at the stage 8b and 9. At stage 10, the DNA fragment signal become very weak in wild type but the *osccrl1* tapetum still has some stronger PCD signal than that in wild type (Fig. 2U, V). At stage 11, no any DNA fragment signal was observed in wild-type tapetal cells, but an obvious TUNEL signal was detected in *osccrl1* tapetal cells (Fig. 2W, X). These results confirm that the tapetum degradation is delayed in the *osccrl1* mutant.

### Map-Based Cloning and Functional Verification of *OsCCRL1*

**Fig. 3:**
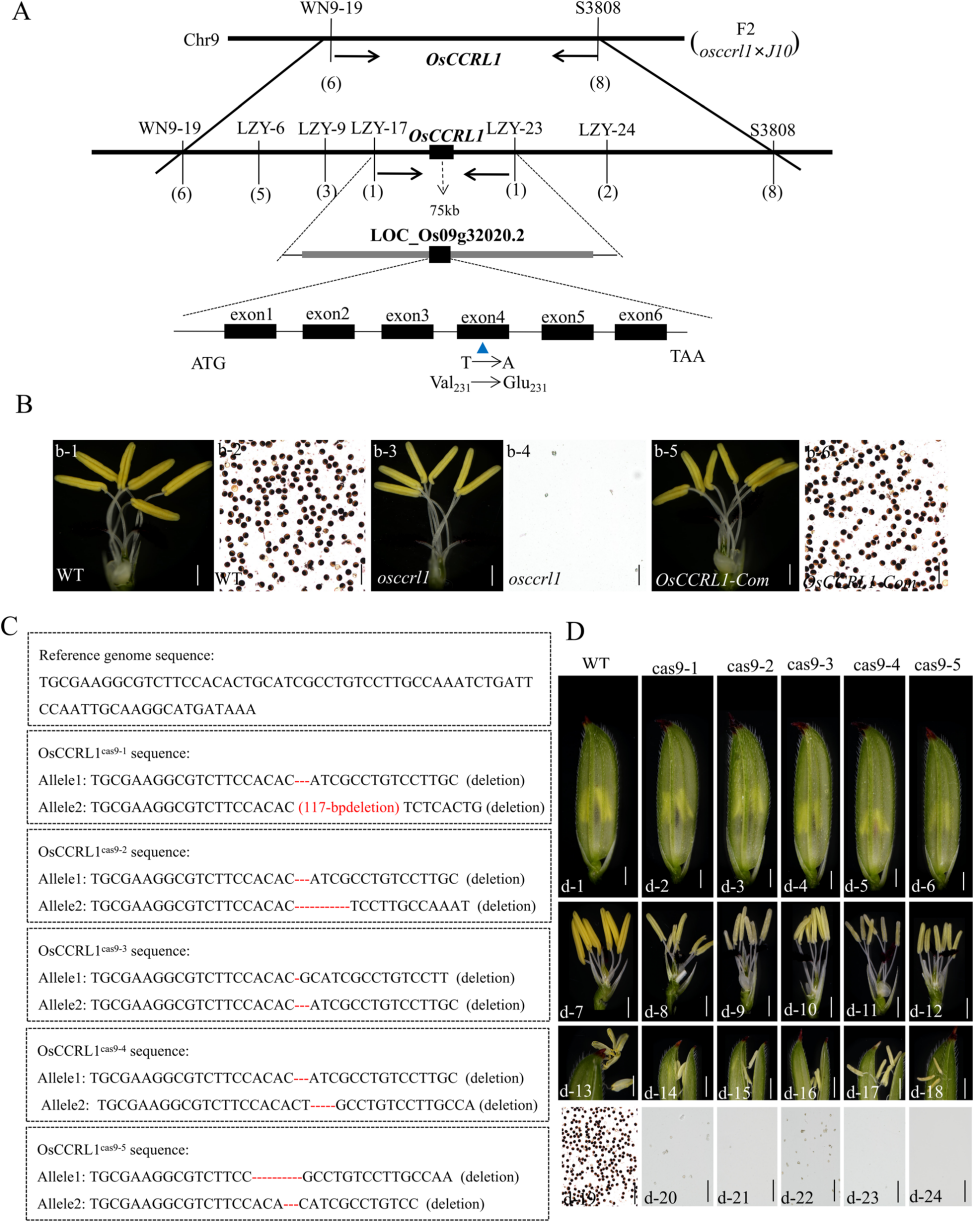
Molecular cloning, complementary validation of *OsCCRL1*, and CRISPR-Cas9-mediated targeted mutagenesis of *OsCCRL1*. **A** Map-based cloning of *OsCCRL1* on chromosome 9. The thick black line represents the chromosome, the markers used for gene mapping are indicated above the black line, numbers in brackets below the black line represent recombinants, and the value below the line represents the genetic distance (cM) between two markers. The *osccrl1* mutant harbors a nucleotide ‘T’ to ‘A’ transition in exon 4, leading to a Val-to-Glu amino acid conversion at site 231. **B** Anther morphology and pollen grains stained by I_2_/KI solution in the wild type, *osccrl1* mutant and *OsCCRL1-*complementary transgenic plant. Scale bars, 2 mm (b-1, b-3 and b-5); 200 μm (b-2, b-4 and b-6). **C** Reference sequence and sequencing of *OsCCRL1*^cas9^ mutant lines. **D** Spikelet, anther, anther dehiscence and pollen grains stained by I_2_/KI solution in wild type and *OsCCRL1*^cas9^ mutant lines. Scale bars, 2 mm (d-1 to d-6); 500 μm (d-7 to d-12); 400 μm (d-13 to d-18); 200 μm (d-19 to d-24)

To determine whether the infertile phenotype of *osccrl1* mutant is controlled by a single gene, we crossed *osccrl1* mutants with the maintainer line ‘Jinhui 10’. All F_1_ individuals exhibited a normal phenotype, and the F_2_ generation showed a segregation ratio of approximately 3:1 (normal:sterile = 387:128, χ2 = 0.0026 < χ2_0.05_ = 3.841), indicating that a single recessive nuclear gene controls this male-sterile phenotype. The *OsCCRL1* locus was mapped by map-based cloning to a 75-kb region flanked by markers LZY17 and LZY23 on chromosome 9. Sequencing analysis identified a single-nucleotide substitution from ‘T’ to ‘A’ in *LOC_Os09g32020*, which was described as *OsTKPR1* (*Tetraketide α-Pyrone Reductase 1*) gene before (Xu et al. 2019). The *LOC_Os09g32020* gene generates two transcripts whose coding sequences do not overlap: *LOC_Os09g32020.*1 with one exon (993 bp) and *LOC_Os09g32020.*2 with 6 exons (1101 bp) (Additional file 1: Fig. S1A, B). The ‘T’ to ‘A’ substitution occurred in the fourth exon of *LOC_Os09g32020.*2 in the *osccrl1* mutant, causing an amino acid conversion from valine (V; a hydrophobic amino acid) to glutamic acid (E; a negatively charged amino acid) at amino acid 231 (Fig. 3A). What needs to be mentioned was that there was one base difference, ‘T’ to ‘G’, in the *LOC_Os09g32020.2* genome between the reference sequence given online and the sequence we sequenced from wild-type DNA (Additional file 1: Fig. S1C), which caused an amino acid change (D to E, aspartic acid to glutamic acid) occurring at the site 80 of OsCCRL1 protein (data not shown).

To verify that the phenotype of the *osccrl1* mutant is caused by the mutation of *LOC_Os09g32020*.2, we introduced a complementation vector containing the normal genomic sequence of the *LOC_Os09g32020*.2 into the *osccrl1* mutant to generate the corresponding transformant *OsCCRL1*-C. Among the 35 transgenic plants generated, 7 complementation-positive plants were obtained. Compared to *osccrl1* mutant and the wild type, the anthers and pollen were restored to the wild-type phenotype in the *OsCCRL1*-C transformants (Fig. 3B).

We then used CRISPR-CAS9 gene editing to knock out *LOC_Os09g32020*.2 in the wild-type cultivar ‘Xinong 1B’ background. Among the 21 transgenic plants generated, 16 knockout-positive plants were obtained. Sequencing revealed five types of mutations among these transgenic plants (Fig. 3C). We observed the 16 transformants throughout the growth period, finding that they were all male sterile. All CRISPR/CAS9 mutant lines grew normal spikelets. When removing the palea and lemma, the anthers of different mutant lines were a bit whiter and shorter than wild-type anthers, but the other floral organs were normal in mutant lines. During the later period of development, when anthers emerge from the glume, wild-type anther filaments grew longer and the anthers protruded from the upper part of the glume. However, the anther filaments of the mutant lines did not seem to grow, and the anthers emerged directly from the dehiscent glumes. Although there were some slight differences in the color of anther (deeper and lighter) and the length of filament (longer and shorter) between different CRISPR/CAS9 mutant lines, the I_2_/KI staining revealed that all of those different mutant lines lacked mature pollen or accumulated little pollen. Therefore, they were infertile (Fig. 3D). These result, coupled with the effect of functional complementation, support the conclusion that *OsCCRL1* is identical to *LOC_Os09g32020*.2*.*

### Expression Analysis and Subcellular Localization of OsCCRL1

**Fig. 4:**
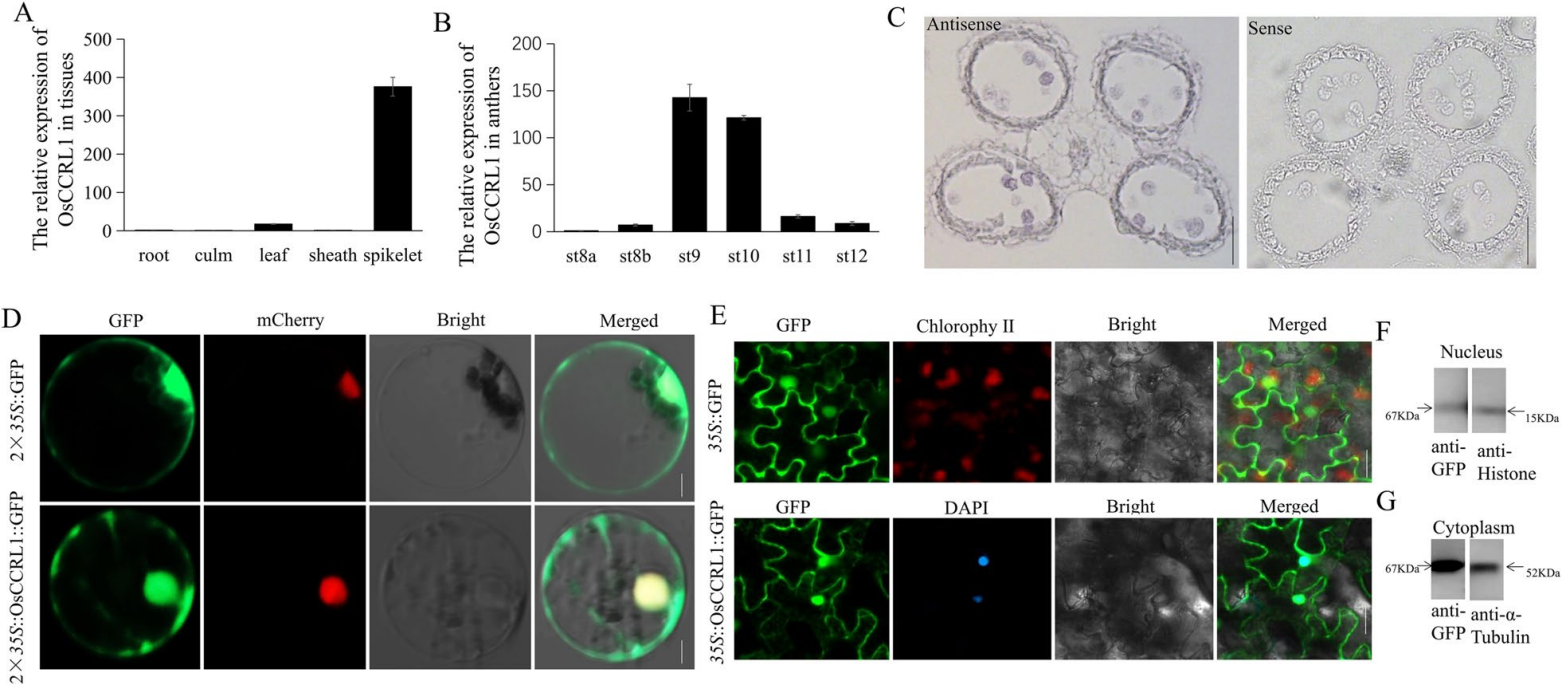
Expression analysis and subcellular localization of *OsCCRL1*. **A**, **B** Abundance of *OsCCRL1* transcripts in wild-type different tissues and anthers at different developmental stages. Data are means ± SD (*n* = 3). **C** mRNA in situ hybridization of *OsCCRL1* transcripts in the wild-type anther. Scale bars, 50 μm. **D** Subcellular localization of 2 × *35S*::GFP and 2 × *35S*::OsCCRL1*-*GFP in rice protoplasts. OsH2B-mCherry was used to indicate the nucleus. Scale bars, 5 μm. **E** Subcellular localization of *35S*::GFP and *35S*::OsCCRL1-GFP with DAPI in *Nicotiana benthamiana.* Scale bar: 5 μm. **F** Immunoblotting of extracted nuclear proteins from tobacco leaves expressing OsCCRL1-GFP using anti-GFP antibodies and Histone antibodies. Histone antibodies was used as the internal reference antibody. **G** Immunoblotting of extracted cytoplasm proteins from tobacco leaves expressing OsCCRL1-GFP using anti-GFP antibodies and Tubulin antibodies. Tubulin antibodies was used as the internal reference antibody.

To examine the expression pattern of *OsCCRL1*, we quantified *OsCCRL1* transcripts in wild-type mature tissues by reverse transcription quantitative PCR (RT-qPCR). The *OsCCRL1* (*LOC_Os09g32020*.2) transcripts were abundant in spikelets, with small amounts in leaves, implied that *OsCCRL1* mainly functions in spikelets (Fig. 4A). At the same time, we found *LOC_Os09g32020*.1 transcript was highly expressed in roots, sheaths, and spikelets, but not culms, indicating that *LOC_Os09g32020*.1 does not function specifically in spikelets (Additional file 1: Fig. S1D). When we performed RT-qPCR in anthers at different developmental stages, *OsCCRL1* transcripts were abundant at stages 9 and 10 relative to the other periods (Fig. 4B) and *LOC_Os09g32020*.1 transcripts were also abundant at stages 9 and 10 (Additional file 1: Fig. S1E). For more detailed analysis of *OsCCRL1* expression, we performed mRNA in situ hybridization of fixed wild-type anthers using digoxygenin (DIG)-labeled antisense and sense probes. Strong DIG signals were observed in tapetal cells and microspores (Fig. 4C). These results confirm that *OsCCRL1* is closely associated with anther and pollen development.

We then transiently expressed GFP driven by two CaMV *35S* promoters and 2 × *35S*::OsCCRL1-GFP fusion protein in rice protoplasts, along with OsH2B-mCherry as a nuclear marker. Using laser confocal microscopy, we detected GFP from the empty vector throughout the protoplast, except the vacuole. By contrast, the GFP signal from 2 × *35S*::OsCCRL1-GFP was located to the cytoplasm and nucleus, partially overlapping the signal with OsH2B-mCherry (Fig. 4D), which was different from the previous reporter that OsTKPR1 was localized in the endoplasmic reticulum (ER) (Xu et al. 2019). Based on this confusion, we transiently expressed the *35S*::OsCCRL1-GFP fusion protein driven by single CaMV*35S* promoter in *Nicotiana benthamiana* leaves and 4',6-diamidino-2-phenylindole (DAPI) was performed to stain the nucleus. In *N. benthamiana* leaves, *35S*::OsCCRL1-GFP localized to the cytoplasm and nucleus, as also observed in protoplasts (Fig. 4E). Finally, to confirm the localization of OsCCRL1, we extracted nuclear and cytoplasmic proteins from *N. benthamiana* leaves expressing *35S*::OsCCRL1-GFP and subjected them to immunoblot analysis with anti-GFP antibody. And OsCCRL1 was identified in nuclear and cytoplasmic extracts from* N. benthamiana* leaves (Fig. 4F, G). These results indicate that OsCCRL1 is expressed in the nucleus and cytoplasm. At the same, we transiently expressed 2 × *35S*::LOC_Os09g32020.1-GFP fusion protein in rice protoplasts. OsH2B-mCherry and OsHDEL-mCherry were used as nuclear and ER markers, respectively. The LOC_Os09g32020.1 protein localized to the ER and the nucleus (Additional file 1: Fig. S1F).

### *OsCCRL1* Affects the Lignin Accumulation and Tapetal PCD

*LOC_Os09g32020*.2 encodes a SDR family enzyme that uses NAD(P)(H) as a co-factor for catalysis. As the plant SDR family is a large family (Moummou et al. 2012), we constructed a phylogenetic tree mainly derived from rice. This phylogenetic analysis suggested that *OsCCRL1* might belong to two enzyme classes, dihydroflavonol-4-reductase (DFR) and cinnamoyl coA reductase (CCR) (Additional file 1: Fig. S2A). DFR is a pivotal oxidoreductase that catalyzes the conversion of dihydroflavonols to generate leucoanthocyanindins during flavonoid biosynthesis (Halbwirth et al. 2006; Petit et al. 2007); CCR catalyzes the hydroxycinnamaldehydes (*p*-coumaraldehyde, coniferaldehyde, and sinapaldehyde) synthesis from hydroxycinnamoyl-CoA thioesters (*p*-coumaroyl-CoA, feruloyl-CoA, sinapoyl-CoA) during lignin biosynthesis (Lacombe et al. 1997; Pan et al. 2014). DFR and CCR may originate from a common ancestor, as indicated by sequence alignment. Plant CCR and DFR, bacterial uridine diphosphate-galactose-4-epimerase (UDP-galactose-4-epimerase) and mammalian 3β-hydroxysteroid dehydrogenase show significant similarities, suggesting that UDP-galactose-4-epimerase might be the common ancestor of CCR and DFR proteins (Lacombe et al. 1997).

The *ostkpr1* mutants, concluding carrying a T-DNA insertion in the second exon of LOC_Os09g32020.2 and a 15-base deletion in the fourth exon of LOC_Os09g32020.2 induced by ^60^Co γ-radiation, lack mature pollen grains (Wang et al. 2013; Xu et al. 2019). Therefore, LOC_Os09g32020.2 was considered to encode tetraketide α-pyrone reductase 1 (TKPR1), a rice ortholog of *Arabidopsis* TKPR1, which plays an important role in the fatty acids and sporopollenin synthesis with acyl-CoA synthetase (ACOS) and polyketide synthase (PKS). TKPR enzymes were initially considered to be DFR-like proteins (Yau et al. 2005; Tang et al. 2009). We therefore constructed a phylogenetic tree of OsCCRL1 based primarily on rice and two *Arabidopsis* TKPR proteins, AtTKPR1 and AtTKPR2. Based on this analysis, OsCCRL1, AtTKPR1, and AtTKPR2 are evolutionarily closer to DFR (Additional file 1: Fig. S2B). We then transiently expressed GFP, *35S*::AtTKPR1-GFP, and *35S*::AtTKPR2-GFP fusion proteins in *N. benthamiana* leaves and performed DAPI staining for the nucleus, and found that AtTKPR1 and AtTKPR2 localized to the cytoplasm and nucleus (Additional file 1: Fig. S3A). We transiently expressed 2 × *35S*::AtTKPR1-GFP and 2 × *35S*::AtTKPR2-GFP in *Arabidopsis* protoplasts. AtVirD2NLS-mCherry was used as a nuclear marker. The GFP signals from 2 × *35S*::AtTKPR1-GFP and 2 × *35S*::AtTKPR2-GFP were located in the cytoplasm and nucleus (Additional file 1: Fig. S3B).

**Fig. 5:**
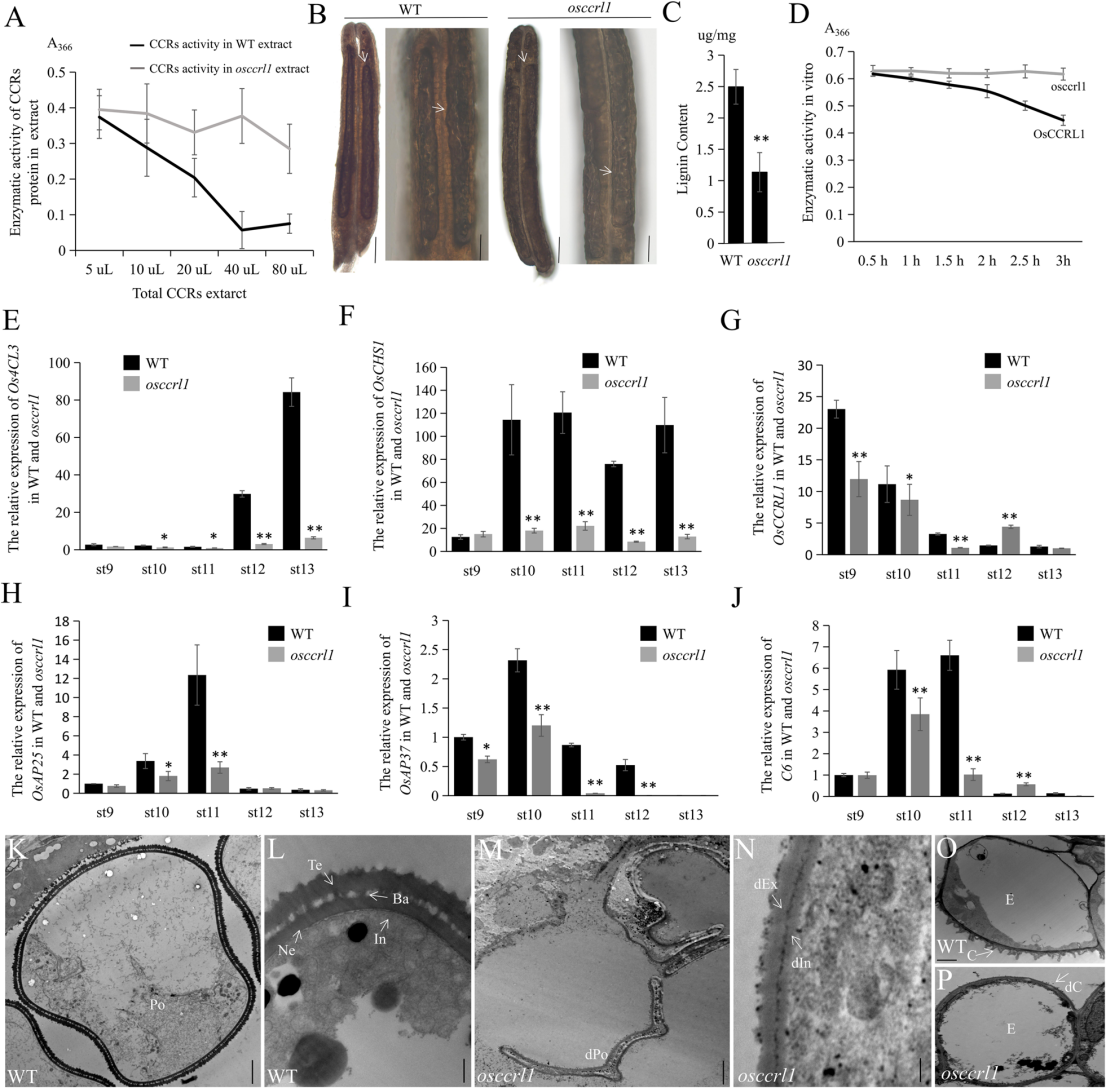
OsCCRL1 process the CCR enzyme activity and is involved in phenylpropane metabolism and tapetum apoptosis. **A** The comparison of extracted CCRs enzyme activity in the wild type and *osccrl1* mutant anthers. Data are means ± SD (*n* = 3). **B** The lignin staining of wild-type and *osccrl1* anthers by phloroglucinol-HCl. Bars, 20 μm (left) and 80 μm (right). **C** The lignin content of wild-type and *osccrl1* spikelets extracted by thioglycolic acid-HCl. Asterisks indicate the significance differences determined by Student’s t-test (*, 0.01 ≤ *P* < 0.05; **, *P* < 0.01). **D** In vitro enzyme activity of OsCCRL1 and osccrl1 proteins. Data are means ± SD (*n* = 3). **E** − **G** The relative expression comparison of *Os4CL3*, *OsCHS1* and *OsCCRL1* in the wild type and *osccrl1* anthers. Data are means ± SD (*n* = 3). Asterisks indicate the significance differences determined by Student’s t-test (*, 0.01 ≤ *P* < 0.05; **, *P* < 0.01). **H** − **J** The relative expression comparison of *OsAP25*, *OsAP37* and *C6* in the wild-type and *osccrl1* anthers. Data are means ± SD (*n* = 3). Asterisks indicate the significance differences determined by Student’s t-test (*, 0.01 ≤ *P* < 0.05; **, *P* < 0.01). **K** − **P** Transmission electron micrograph of pollen wall and anther cuticle in the wild type and *osccrl1* mutant. Po, pollen; Te, tectum; Ba, bacula; Ne, nexine; In, intine; dPo, defective pollen; dIn, defective intine; dEx, defective exine; E, epidermis; C, cuticle; dC, defective cuticle. Scale bars, 1 μm in (k, m), 0.2 μm in (l, n), and 2 μm in (o, p)

In previous study, the *Arabidopsis* DFR (*tt3*) null mutation could not be complemented by *35S::AtDRL1/AtTKPR1* gene, and standard DFR substrates (dihydrokaempferol and dihydroquercetin) could not be metabolized by *N. tabacum* (Nt) TKPR1 or OsTKPR1 in vitro (Tang et al. 2009; Wang et al. 2013). For this, a conclusion that TKPR1 were unlikely to process the activity of DFR enzymes participating in flavonoid biosynthesis was decided (Petit et al. 2007). In our study, we quantified the transcript level of *OsPKS2* (*Polyketide Synthase 2*), which is involved in fatty acid biosynthesis with OsTKPR1, in wild-type and *osccrl1* anthers by RT-qPCR, and observed no significant differences of *OsPKS2* expression between wild-type and *osccrl1* mutant (Additional file 1: Fig. S4A). Given this and previous findings, we investigated whether LOC_Os09g32020.2 was invovled in lignin synthesis. First, we extracted total CCRs from wild-type and *osccrl1* anthers and performed an assay using ferulyl-CoA in vitro according to a published procedure (Goffner et al. 1994). When we measured the UV absorption of the reaction mixture at 366 nm after incubation, the absorption of wild-type CCRs decreased more rapidly than that from the *osccrl1* mutant (Fig. 5A), implying that the *osccrl1* mutant has reduced CCRs activity. CCR is a limiting enzyme with a key role in lignin biosynthesis. We therefore examined lignin levels in wild-type and *osccrl1* anthers upon phloroglucinol-HCl staining (Hao et al. 2014). Wild-type anthers had darker staining than that of *osccrl1* mutant, indicative of higher lignin contents than *osccrl1* anthers (Fig. 5B). The lignin of wild-type and *osccrl1* anthers was extracted by thioglycolic acid-HCL and dissolved in NaOH, and it was found that the lignin content of wild-type anthers was relatively higher than that of *osccrl1* mutant (Fig. 5C). Next, the enzyme activity of recombinant protein OsCCRL1 and osccrl1 was assayed in vitro using with ferulyl-CoA substrate. The UV absorption of the reaction mixture at 366 nm after incubation of OsCCRL1 decreased more rapidly than that of osccrl1 (Fig. 5D). These indicated that OsCCRL1 participated in the synthesis of lignin.

Based on this finding, we named the *LOC_Os09g32020.2* gene as *cinnamoyl coA reductase-like 1* (*OsCCRL1*). Because CCR also plays an important role in the shunting of components in the phenylpropanoid pathway, we examined the expression of *Os4CL3* (*4-Coumarate: coenzyme A ligase 3*), *OsCHS1* (*chalcone synthase 1*), and *OsCCRL1* in wild-type and *osccrl1* anthers, which are regarded to associate with phenylpropanoid pathway. The levels of all these transcripts were reduced in *osccrl1* mutant (Fig. 5E–G), indicating that the mutation of *OsCCRL1* also affects the phenylpropanoid metabolism. As normal tapetal PCD was also delayed in *osccrl1* anther revealed by TUNEL assays above, we measured the transcript levels of some key genes involved in tapetal PCD, finding that *OsAP25* (*Aspartic Protease 25*), *OsAP37* (*Aspartic Protease 37*), and *OsC6* transcript levels were also strongly reduced in *osccrl1* mutant (Fig. 5H–J). Tapetum cells could secrete sporopollenin precursors for the formation of pollen exine, and the sporopollenin synthesis and pollen intine formation requires the participation of phenylpropanoid metabolites. In *osccrl1* mutant, tapetum development was delayed and phenylpropane metabolism was disordered, which suggested that the pollen wall formation of *osccrl1* mutants might be affected. Therefore, the wild-type and *osccrl1* mutant pollen wall were observed by transmission electron microscopy (TEM), and it was found that the wild-type pollen had a complete pollen wall structure, including tectum, bacula, nexine and intine (Fig. 5K, L). However, the pollen wall structure of *osccrl1* mutant was abnormal. In *osccrl1* mutant, the bacula was missing, causing tectum and nexine fused together to form a thinner and defective exine. In addition, the pollen intine of *osccrl1* mutant was also thinned (Fig. 5M, N). The synthesis of cutin and wax also requires the phenylpropane metabolites. Scanning electron microscopy found that the anthers epidermis of *osccrl1* mutant was smoother and defective. When observing the pollen wall by TEM, we also found obvious differences between the surface of wild-type and *osccrl1* mutant anther epidermis. A protruding structure, anther cuticle, composed of cutin and wax was found in the wild type, but the *osccrl1* mutant lacked this (Fig. 5O, P).

### OsCCRL1 is Directly Regulated by OsMYB103

**Fig. 6:**
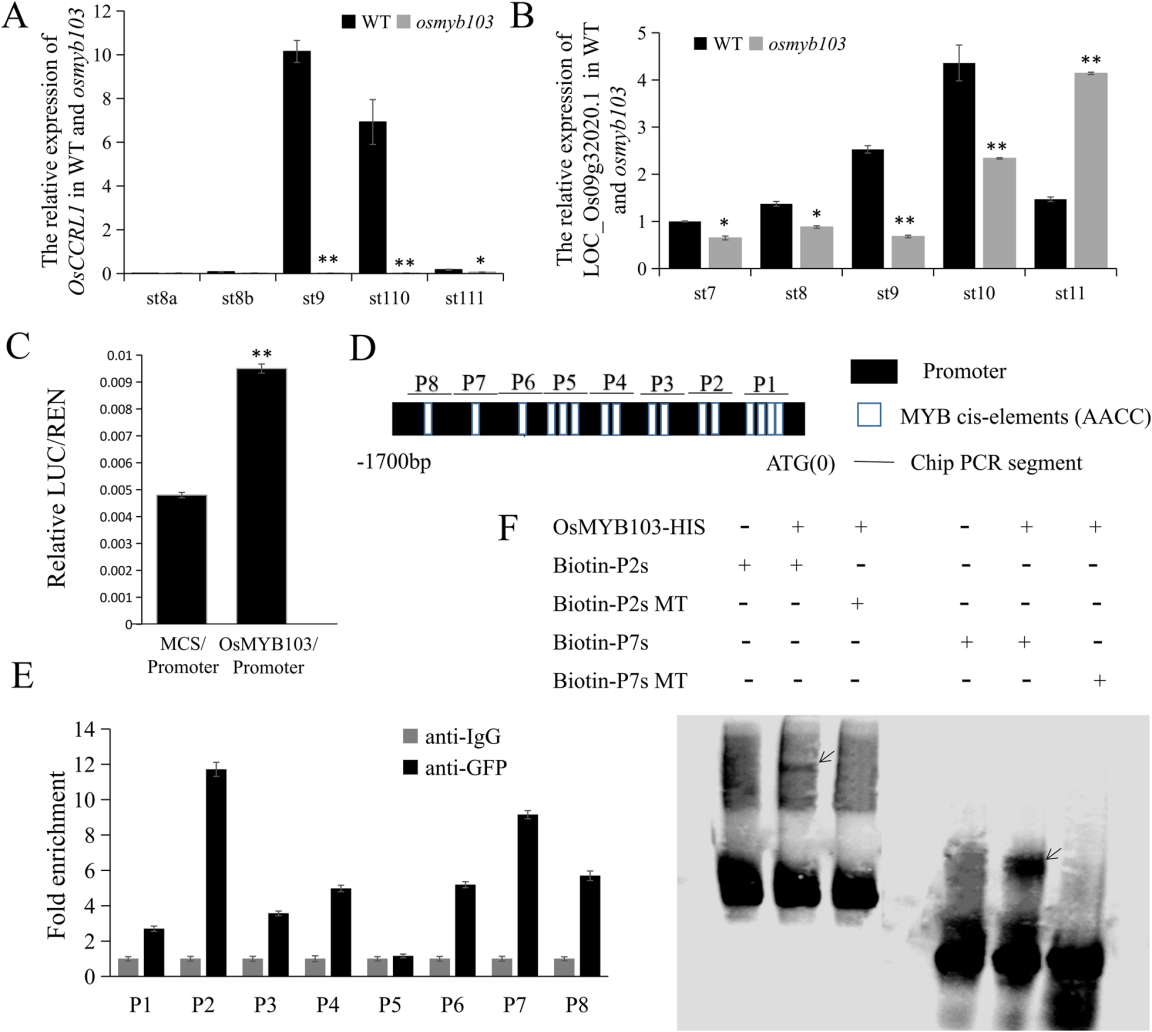
OsMYB103 regulates the expression of *OsCCRL1.* **A**, **B** The relative expression comparison of *OsCCRL1* and *LOC_Os09g32020.1* between the wild-type and *osmyb103* mutant anthers. Data are means ± SD (*n* = 3). Asterisks indicate the significance differences determined by Student’s t-test (*, 0.01 ≤ *P* < 0.05; **, *P* < 0.01). **C** Transient expression assay of the LUC reporter gene with the multiple cloning site (MCS) effector plasmid using as a control. Data are means ± SD (*n* = 3). Asterisks indicate the significance differences determined by Student’s t-test (*, 0.01 ≤ *P* < 0.05; **, *P* < 0.01). **D** Schematic diagrams of the distribution of ‘AACC’ motifs (*cis*-elements recognized by OsMYB103 transcription factors) in the OsCCRL1 promoter regions. **E** A ChIP-qPCR analysis of OsMYB103 binding the ‘AACC’ motifs to *OsCCRL1* promoter regions in *Ubi::OsMYB103-GFP* plants with anti-GFP antibody. Data are means ± SD (*n* = 3). **F** An EMSA assay of OsMYB103 protein directly binding to the probe ‘P2s’ (93 bp) and ‘P7s’ (40 bp) of OsCCRL1 promoter. ‘Biotin-P2s mutant’ and ‘Biotin-P7s mutant’ mean the mutant probes at ‘AACC’ motifs

Given that *OsCCRL1* is expressed in a relatively spatiotemporally specific pattern, we then investigated the precise regulation of it. Various MYB transcription factors, the MBW ternary complex, and additional transcription factors are key transcriptional regulators of phenylpropanoid metabolism, especially lignin and flavonoid biosynthesis (Zhong et al. 2010; Feller et al. 2011; Hichri et al. 2011; Liu et al. 2015; Xu et al. 2015; Yang et al. 2017; Ma and Constabel 2019). In *Arabidopsis*, many MYB transcription factors function in the biosynthesis of secondary cell wall components, including cellulose, xylan, and lignin, such as MYB15, MYB46, MYB52, MYB54, MYB103, MYB85, MYB43, and MYB20 (Zhong et al. 2008; Chezem et al. 2017; An et al. 2019). In rice, OsMYB30 binds to and activates the *OsPAL6* (*Phenylalanine Ammonia-Lyase 6*), *OsPAL8* (*Phenylalanine Ammonia-Lyase 8*), *Os4CL3* (*4-Coumarate: coenzyme A Ligase 3*), and *Os4CL5* (*4-Coumarate: coenzyme A Ligase 5*) promoters to regulate phenylpropanoid metabolism (He et al. 2020; Li et al. 2020). OsMYB103 is an important R2R3 MYB transcription factor that positively regulates tapetum degradation (Zhang et al. 2010; Pan et al. 2020; Xiang et al. 2020; Han et al. 2021; Lei et al. 2022), and different type mutation of OsMYB103 in several different rice were male sterile. We previously identified the *osmyb103* mutant (Additional file 1: Fig. S4B). In addition to delayed PCD in the tapetum, the *osmyb103* mutant shows whitish anthers, abnormal sporopollenin synthesis, lack of Ubisch bodies, smoother anther epidermis, and defective pollen (Lei et al. 2022), suggesting that it may regulate phenylpropanoid metabolism. To investigate whether *OsCCRL1* is regulated by OsMYB103, *OsCCRL1* expression was detected by RT-qPCR in the wild type and *osmyb103* anther at first [This *osmyb103* mutant was isolated and identified in our previous study (Lei et al. 2022)]. The expression of *OsCCRL1* almost none was detected in *osmyb103* mutant (Fig. 6A), indicating that mutation of *OsMYB103* affected the expression of *OsCCRL1.* We also examined the expression of *LOC_Os09g32020.1* in the wild type and *osmyb103* anther, finding no significant difference between them (Fig. 6B). These results, combined with the results of the subcellular localization analysis, suggest that the *LOC_Os09g32020.1* and *LOC_Os09g32020.2* mRNAs are two different transcripts.

Then, OsMYB103 could activate the *OsCCRL1* promoter in a dual luciferase assay in rice protoplasts, with about three-fold higher LUC/REN ratio compared to the control (Additional file 1: Fig. S4C; Fig. 6C), suggesting that the *OsCCRL1* promoter is regulated by OsMYB103. To confirm this notion, we performed chromatin immunoprecipitation and quantitative-PCR (ChIP-qPCR) analysis in *Ubi::OsMYB103-GFP* transgenic anthers. We detected 16 ‘AACC’ *cis*-elements recognized by OsMYB103 in the *OsCCRL1* promoter within a 1700-bp region upstream of the transcription start site (Fig. 6D). Using primers containing the ‘AACC’ *cis-*element designed based on the *OsCCRL1* promoter, all regions except ‘P5’ were enriched in *Ubi::OsMYB103-GFP* transgenic anthers with anti-GFP antibody (Fig. 6E). To further confirm that OsMYB103 directly binds the *OsCCRL1* promoter, we performed an electrophoretic mobility-shift assay (EMSA) and found that OsMYB103 bound directly to the ‘P2s’ and ‘P7s’ probes of *OsCCRL1* promoter (Fig. 6F). These results confirm that OsMYB103 directly regulates *OsCCRL1* expression.

### Phenotypic Analysis of *Osmyb103 osccrl1* Double Mutant

**Fig. 7:**
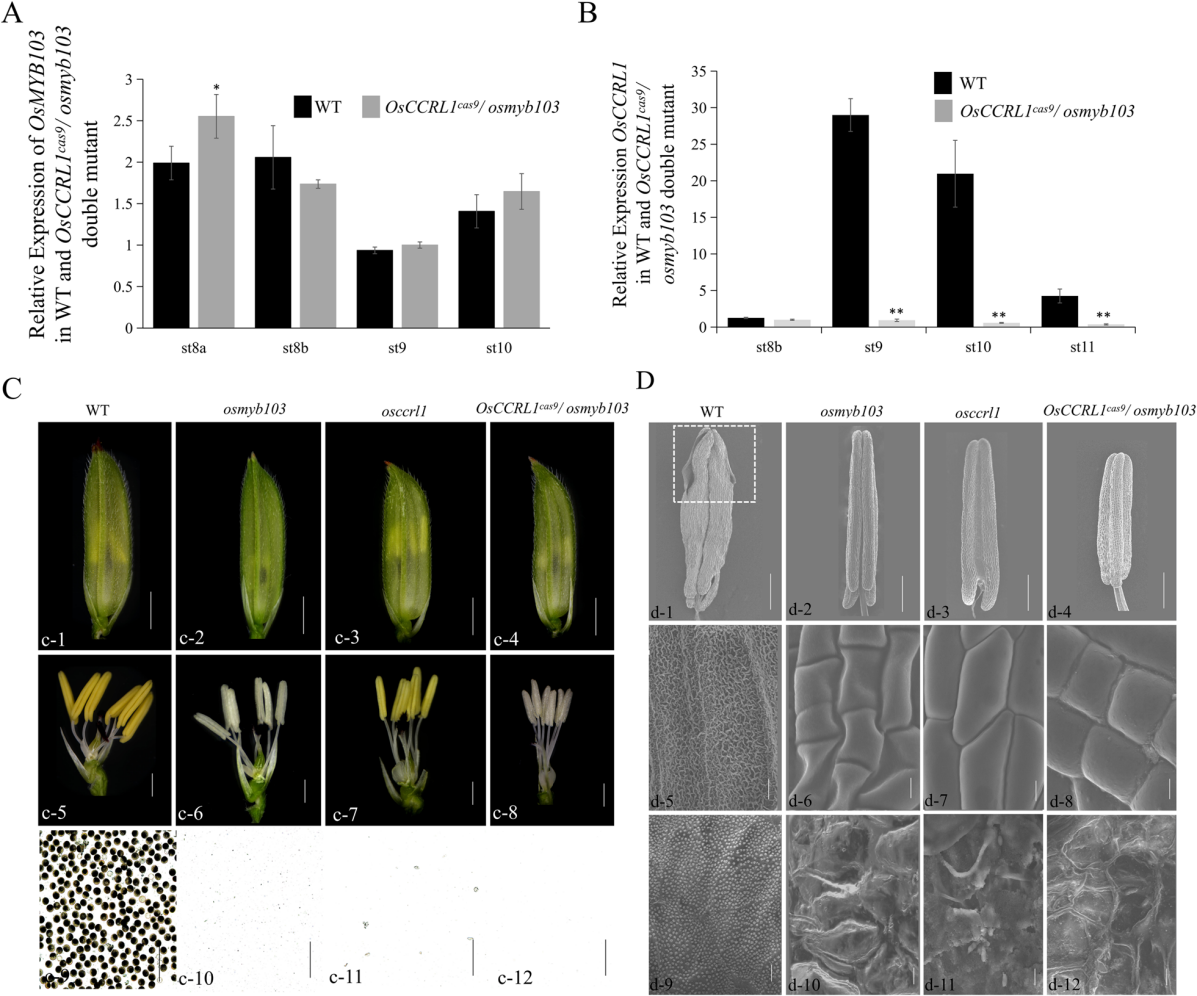
Phenotypic analysis of *osmyb103 osccrl1* double mutant transgenic plants. **A**, **B** The relative expression comparison of *OsMYB103* and *OsCCRL1* between the wild-type and *osmyb103 osccrl1* double mutant anthers. Data are means ± SD (*n* = 3). Asterisks indicate the significance differences determined by Student’s t-test (*, 0.01 ≤ *P* < 0.05; **, *P* < 0.01). **C** Spikelet, anther and pollen stained by I2/KI solution of wild type, *osmyb103* mutant, *osccrl1* mutant, and *osmyb103 osccrl1* double mutant. Scale bars, 2 mm (c-1 to c-4); 500 μm (c-5 to c-8); 200 μm (c-9 to c-12). **D** The scanning electron microscopy of anther, anther epidermis surface, anther inner surface in wild type, *osmyb103* mutant, *osccrl1* mutant, and *osmyb103 osccrl1* double mutant. Scale bars, 200 μm (d-1 to d-4); 50 μm (d-5 to d-12)

Taking into account the relationship between OsMYB103 and OsCCRL1, we generated the *osmyb103 osccrl1* double mutant by introducing the *OsCCRL1* knockout vector into the *osmyb103* mutant. We identified *osmyb103 osccrl1* double mutant transgenic plants by sequencing (Additional file 1: Fig. S4D, E) and measured *OsMYB103* and *OsCCRL1* transcript levels in wild-type and *osmyb103 osccrl1* double mutant anthers by RT-qPCR. *OsMYB103* transcript levels did not significantly differ between two lines (Fig. 7A), but *OsCCRL1* was not expressed at all in *osmyb103 osccrl1* double mutant anthers (Fig. 7B), implying that OsMYB103 functions upstream of OsCCRL1. When we compared the phenotypes of the *osmyb103* mutant, *osccrl1* mutant and *osmyb103 osccrl1* double mutants, the phenotype of *osmyb103 osccrl1* double mutant was closer overall to that of *osmyb103* single mutant, although in some respects the phenotypes of *osmyb103* and *osccrl1* were not easily distinguishable. For example, all three mutants lacked pollen, none showed anther dehiscence, and all had severely defective anther epidermis and inner surface (Fig. 7C, D).

**Fig. 8:**
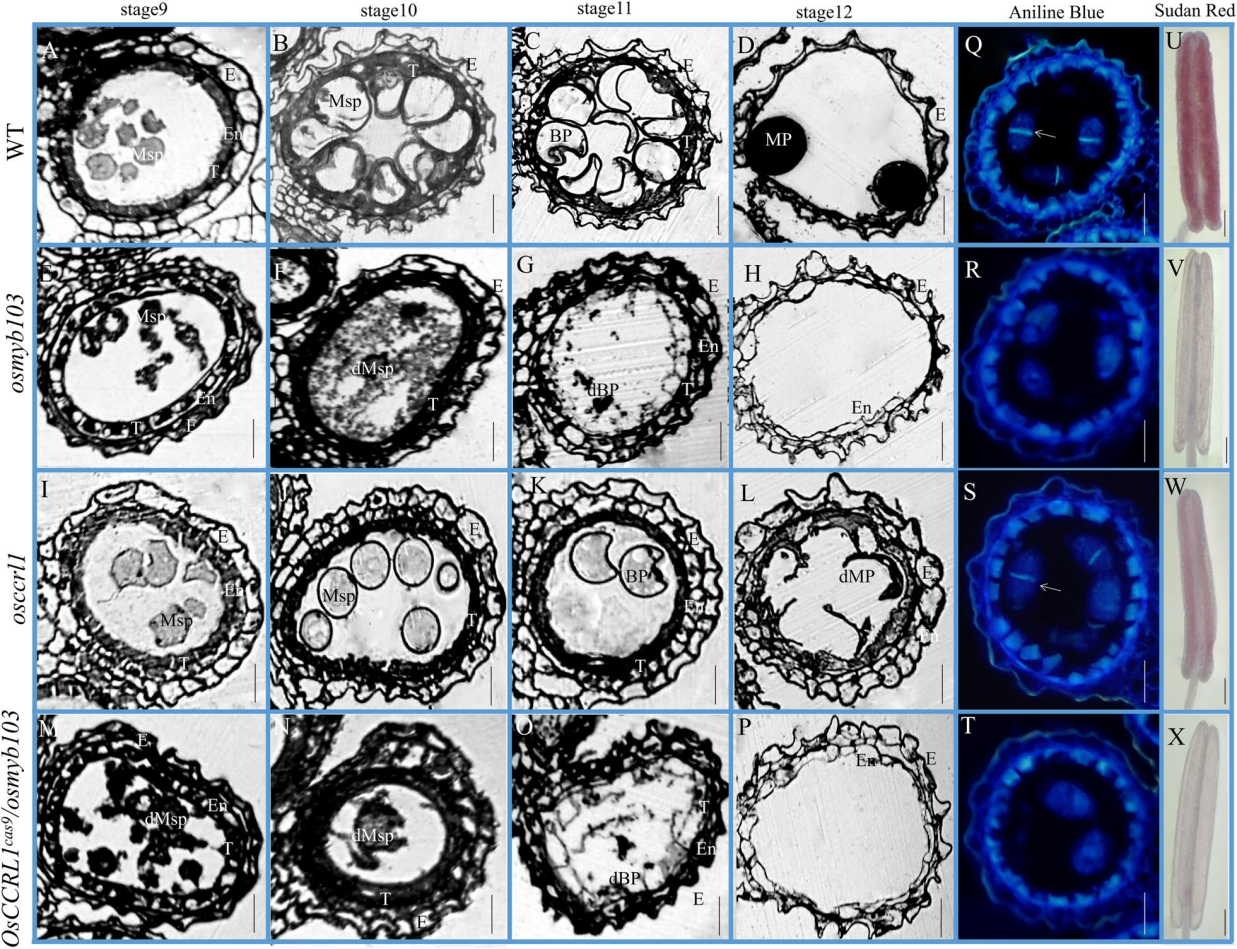
Semithin section, aniline staining and sudan red staining of the wild type, *osmyb103*, *osccrl1*, and *osmyb103 osccrl1* double mutants. **A**–**P** Semi-thin sections of anthers at stages 9, 10, 11 and 12 in the wild type, *osmyb103* mutant, *osccrl1* mutant, and *osmyb103 osccrl1* double mutant. The images show a single locule of an anther. E, epidermis; En, endothecium; T, tapetum; Msp, microspore; BP, bicellular pollen; MP, mature pollen; dMP, mature pollen. Scale bars, 25 μm. **Q**–**T** Callose staining of the wild type, *osmyb103* mutant, *osccrl1* mutant, and *osmyb103 osccrl1* double mutant anther at stage 8b. The images show a single locule of an anther. Scale bars, 25 μm. **U**–**X** Sudan red of the wild type, *osmyb103* mutant, *osccrl1* mutant, and *osmyb103* osccrl1 double mutant anther. Scale bars, 200 μm

Therefore, semi-thin sections were performed and it showed that the abnormality of *osmyb103 osccrl1* double mutant anthers began to be detectable at stage 9. During this stage, the tapetum of the double mutant began to swell, resembling the phenotypes of the *osmyb103* mutant. At stage 11, the abnormality of the microspores appeared to be more serious in the double mutant than in *osccrl1*, such as the abortion phenotype of microspores, resembling the phenotype of the *osmyb103* mutant (Fig. 8A–P). To identify the phenotype of *osmyb103 osccrl1* double mutant was closer to *osmyb103* mutant, we performed aniline blue staining to observe anther callose at stage 8b, observing a clear callose signal in the wild type and *osccrl1* mutant, but not in *osmyb103* mutant or *osmyb103 osccrl1* double mutant (Fig. 8Q–T). Then, we stained anthers with sudan red 7B to identify the fatty acid differences. The anthers of the *osmyb103* mutant, *osccrl1* mutant, and *osmyb103 osccrl1* double mutant showed less sudan red 7B staining than those of the wild type and the staining pattern of the *osmyb103 osccrl1* double mutant was closer to that of *osmyb103* mutant (Fig. 8U–X) again suggesting that OsCCRL1 functions downstream of OsMYB103.

## Discussion

### The Potential Role of OsCCRL1 in Phenylpropane Metabolism

In flowering plants, environmental changes easily affect the developing male gametophytes. To cope with this issue, plants have evolved two protective structures, the anther cuticle and pollen wall, composed of lipidic and phenolic compounds. Therefore, anther and pollen development requires the accumulation of large amounts of phenylpropanoid metabolites, especially lignin, hydroxycinnamic acid amides, and flavonol glycosides. Changes in the composition and levels of phenylpropanoids can affect anther development and pollen wall patterning (Herdt et al. 1978; Fellenberg and Vogt 2015; Shi et al. 2015). *Arabidopsis REF3* gene encodes C4H, which transforms *para*-hydroxylates cinnamic acid to *p*-coumaric acid in the phenylpropanoid pathway. *P*-coumaric acid ester was considered to be the precursor of sporopollenin and the *ref3-2* mutant produces little mature pollen and indehiscent anthers. The sterile phenotype of *ref3-2* is due to the decrease in C4H enzyme activitywhich leads to reduced *p*-coumaric acid ester and ultimately decreased sporopollenin production (Schilmiller et al. 2009). In the current study, we identified the male-sterile rice mutant *osccrl1* from *indica*, with abnormal anther cuticles and Ubisch bodies, as revealed by cytological observation, along with seriously defective pollen wall and tapetum development. Our phenotypic analyses indicated that OsCCRL1 has a comprehensive effect on anther development, especially on anther cuticle, pollen wall, Ubisch bodies and anther dehiscence, which are somehow related to phenylpropanoid metabolism.

Previous studies had identified *LOC_Os09g32020.2* as OsTKPR1, which was considered to function in the fatty acid metabolic pathway for sporopollenin synthesis and pollen exine formation with ACOS and PKSA/B (Xu et al. 2019; Wang et al. 2013). *ostkpr1-2*, which was completely male sterile with smaller and paler anthers containing no viable pollen grains, was screened from a radiated mutant library by ^60^Co γ-ray of 9522 (*O. sativa ssp japonica*) and showed a 15 nucleotide deletion in the fourth exon of LOC_Os09g32020.2 (Xu et al. 2019). Our mutant *osccrl1* from *indica* was also completely male sterile without any viable pollen grains and showed one base substitution in the fourth exon of LOC_Os09g32020.2. Combining *ostkpr1-2* and *osccrl1* mutants, it showed that LOC_Os09g32020.2 controlled the anthers fertility both in *japonica* and *indica*. The difference between *ostkpr1-2* and *osccrl1* mutants was that the anthers of *ostkpr1-2* were smaller and paler, and the abnormality of anthers in *ostkpr1-2* began at the 9 stage, but the anther of *osccrl1* mutant did not show smaller and whiter, and the abnormality of anthers in *osccrl1* began at the 10 stage, which may be caused by their mutation sites, causing *ostkpr1-2* mutant more serious.

Our phylogenetic analysis classified LOC_Os09g32020.2 into both the DFR family (involved in flavonoid biosynthesis) and the CCR family (involved in lignin biosynthesis). Through phylogenetic analysis, AtTKPR1 and AtTKPR2, two *Arabidopsis* homologs of LOC_Os09g32020.2, also belong to the DFR family. Therefore, combining previous reporters with our finding, OsTKPR1/OsCCRL1 might be involved fatty acid metabolism, flavonoid biosynthesis, and lignin biosynthesis. However, previous studies have shown that OsTKPR1 might not have a DFR-like function (Tang et al. 2009). And the expression of *OsPKS2*, thought to be involved in the same metabolic pathway with OsTKPR1/OsCCRL1, was not significantly altered in *osccrl1* anther. The expression of OsCCRL1 itself in *osccrl1* mutant was reduced, but the expression of *OsPKS2* in *osccrl1* mutant did not change much, suggesting that OsCCRL1 may be involved in another pathway which differed with *OsPKS2*. Based on these findings, we focused on the role of LOC_Os09g32020.2 as a CCR enzyme. Then, biochemical analysis revealed that total CCRs activity was higher in wild-type anthers than that in *osccrl1* anthers, and lignin accumulation and content was reduced in *osccrl1* anther. In addition, the recombinant protein OsCCRL1 may have CCR enzymatic activity in vitro, but the osccrl1 lost it. CCR is considered to bind the coenzymes NAD(P)(H) at the *N*-terminal region, and bind the substrate at the *C*-terminal region during the catalytic reaction. Therefore, we guess that the substitution of valine (hydrophobic) to glutamic acid (negatively charged) of OsCCRL1 possibly altered the affinity of OsCCRL1 to the substrate, further affecting the binding to the substrate. And eventually, the osccrl1 losing the CCR enzymatic activity.

CCR is a key enzyme in the phenylpropanoid pathway. *Os4CL3* and *OsCHS1*, two other key enzymes in this pathway, were also greatly reduced in *osccrl1* anthers, suggesting that the phenylpropanoid pathway was broadly disrupted. Phenylpropanoid pathway is also altered in *ostkpr1-2* mutants (Xu et al. 2019). Therefore, OsTKPR1/OsCCRL1 plays an important role in the phenylpropanoid pathway. And it might be the disturbed phenylpropanoid pathway in the *osccrl1* mutant that blocked the several components formation, such as cuticles, sporopollenin and lignin, further resulting in pollen infertility and anther indehiscence.

It is worth noting that the previously reported OsTKPR1 was located in the endoplasmic reticulum in tobacco leaf cells, but OsCCRL1 was localized in cytoplasm and nucleus in rice protoplasts and tobacco leaf cells in our study. We also found that in previous reports, AtTKPR1 was located in endoplasmic reticulum and AtTKPR2 was located in cytoplasm (Grienenberger et al. 2010; Lallemand et al. 2013). But DRL1 (Dihydroflavonol 4-reductase-like 1), that was AtTKPR1, was located in cytoplasm and nucleus (Tang et al. 2009). Therefore, TKPRs may be located in endoplasmic reticulum, nucleus and cytoplasm. What caused the subcellular localization difference of OsTKPR1 and OsCCRL1? We noticed that there was one base difference in the LOC_Os09g32020.2 genome sequence between the reference sequence given online and the sequence we sequenced from wild-type DNA, meaning that there was an amino acid difference at the site 80 between the online sequence and OsCCRL1. At the same time, the amino acid at the site 80 of protein OsTKPR1 was aspartic acid (D), which was consistent with the one online. Therefore, it may be this difference leads to the pattern change of subcellular localization between OsTKPR1 and OsCCRL1.

### OsCCRL1 was Directly Regulated by OsMYB103

The MYB transcription factors is highly conserved and could activate or inhibit downstream gene expression. Various MYB transcription factors have been shown to be required for the regulation of phenylpropanoid metabolism. In rice, *OsPAL6* and *OsPAL8* are directly upregulated by OsMYB30, and OsMYB30 also binds to the promoters of *Os4CL3* and *Os4CL5*, leading to guaiacyl lignin and syringyl lignin accumulation (He et al. 2020; Li et al. 2020). In addition, MYB15 directly binds to the promoters of *PAL1*, *C4H*, and *COMT* to promote guaiacyl lignin biosynthesis (Chezem et al. 2017). LTF1, a MYB transcriptional repressor, represses lignin biosynthesis by binding to the promoter of *4CL*, whereas the degradation of LTF1 via the proteasome pathway activates lignin biosynthesis (Gui et al. 2019).

*OsMYB103*/*OsMYB80*/*OsMS188/BM1* encodes an R2R3 MYB transcription factor that is highly conserved in both dicot and monocot plants. In *japonica* and *indica* rice, EMS-treated induced mutants and knockout transgenic plants of *OsMYB103*/*OsMYB80*/*OsMS188/BM1* are completely male sterile, with small and white anthers. These mutants exhibit a lack of Ubisch bodies, and no mature microspores, and delayed tapetum PCD (Zhang et al. 2010; Xiang et al. 2020; Han et al. 2021; Lei et al. 2022), indicating that OsMYB103/OsMYB80/OsMS188*/BM1* plays an important role in tapetum and pollen development, and this gene was easily induced to mutate. Although the *osmyb103* mutants obtained by different methods were slightly different in some details, such as anther anthers epidermis, tapetum thickness, and the stage of abnormal anther, these *osmyb103* mutants were all male sterile. OsMYB103/OsMYB80/OsMS188*/BM1* transcription factor directly binds to the promoter regions of *CYP703A3*, *CYP704B2*, *OsPKS2*, *OsPKS1*, *DPW*, *ABCG15*, *EAT1*, and *PTC1* to regulate their expressions (Xiang et al. 2020; Han et al. 2021; Lei et al. 2022). Comparative transcriptome analysis (RNA-seq) of downstream differentially expressed genes (DEG) in wild type and *osmyb80* mutant*,* it suggested that OsMYB103/OsMYB80/OsMS188*/BM1* functions in many processes, such as fatty acid and small-molecule metabolic process, transcriptional regulation process, ubiquitin-dependent protein degradation process, oxidation and reduction process, binding processes with proteins, lipids, and coenzymes, and oxidoreductase and hydrolase activity (Pan et al. 2020). Therefore, OsMYB103/OsMYB80/OsMS188*/BM1* is a crucial transcription factor that regulates the expression of many downstream genes and participates in many pathways to function in anther and pollen development. OsMYB103/OsMYB80/OsMS188/BM1 might also be involved in regulating the phenylpropanoid pathway and lignin biosynthesis.

In our study, *OsCCRL1* expression was significantly reduced in *osmyb103* anthers. A dual luciferase assay in rice protoplasts, a ChIP analysis in *Ubi::OsMYB103-GFP* transgenic plants, and an EMSA analysis with OsMYB103 protein confirmed that OsMYB103 binds to the *OsCCRL1* promoter to regulate its expression. More importantly, compared with the individual mutants *osccrl1* and *osmyb103*, some phenotypes of the *osmyb103 osccrl1* double mutant were biased towards those of *osmyb103* mutant, such as whiter anthers, abnormal anther morphology at stage 9, lack of callose accumulation in microspores at stage 8b, and reduced sudan red staining of anthers. In the *osmyb103 osccrl1* double mutant, *OsCCRL1* expression was drastically reduced, but *OsMYB103* expression seemed to be unchanged. Therefore, OsMYB103 is located upstream of *OsCCRL1* and binds to its promoter to regulate its expression, which showed OsMYB103 also plays an important role in phenylpropanoid metabolism and tapetum development in rice anthers.

## Conclusion

In this study, we demonstrated that OsCCRL1 (LOC_Os09g32020.2), one member of SDR, is involved in the phenylpropanoid pathway in rice anthers. *OsCCRL1* is specifically expressed in the tapetum and microspores, and a replacement of valine (hydrophobic) by glutamic acid (negatively charged) at position 231 of *OsCCRL1* led rice to have abnormal anthers, delayed tapetum development, reduced lignin accumulation, and complete male sterility. Solid genetic evidences, including genetic complementation and CRISPR/CAS9-derived transgenic plants analysis, supported the notion that *OsCCRL1* is required for male fertility. Besides, OsCCRL1 was contributed to phenylpropanoid metabolism, which is vital for pollen wall formation, anther cuticle development, and anther dehiscence. In addition, we showed that *OsCCRL1* is directly regulated by OsMYB103/OsMYB80/OsMS188*/BM1*, as confirmed by analysis of the *osmyb103 osccrl1* double mutant, revealing a new regulatory pathway of phenylpropanoid metabolism and tapetal development in rice anthers.

## Supplementary Information

**Additional file 1. Fig. S1** Two LOC_Os09g32020 transcripts, expression analysis and subcellular localization of LOC_Os09g32020.1. **Fig. S2** Phylogenetic tree of OsCCRL1. **Fig. S3** Subcellular localization of two Arabidopsis TKPRs. **Fig.S4** The relative expression of OsPKS2, the schematic diagrams of transient expression assay and the sequencing of osmyb103 osccrl1 double mutant.

## Data Availability

The data that support the findings of this study are available from the corresponding author N.W. and G.H, upon reasonable request.
