# Supplementary material for: OsCCRL1 is Essential for Phenylpropanoid Metabolism in Rice Anthers
Source: Rice (N Y). 2023 Feb 27;16:10. doi: 10.1186/s12284-023-00628-1 (PMC9971536; doi:10.1186/s12284-023-00628-1)
Supplement: Supplementary file 1 — Additional file 1. Fig. S1 Two LOC_Os09g32020 transcripts, expression analysis and subcellular localization of LOC_Os09g32020.1. Fig. S2 Phylogenetic tree of OsCCRL1. Fig. S3 Subcellular localization of two Arabidopsis TKPRs. Fig.S4 The relative expression of OsPKS2, the schematic diagrams of transient expression assay and the sequencing of osmyb103 osccrl1 double mutant. [file 12284_2023_628_MOESM1_ESM.doc]

**Supplemental Figures**


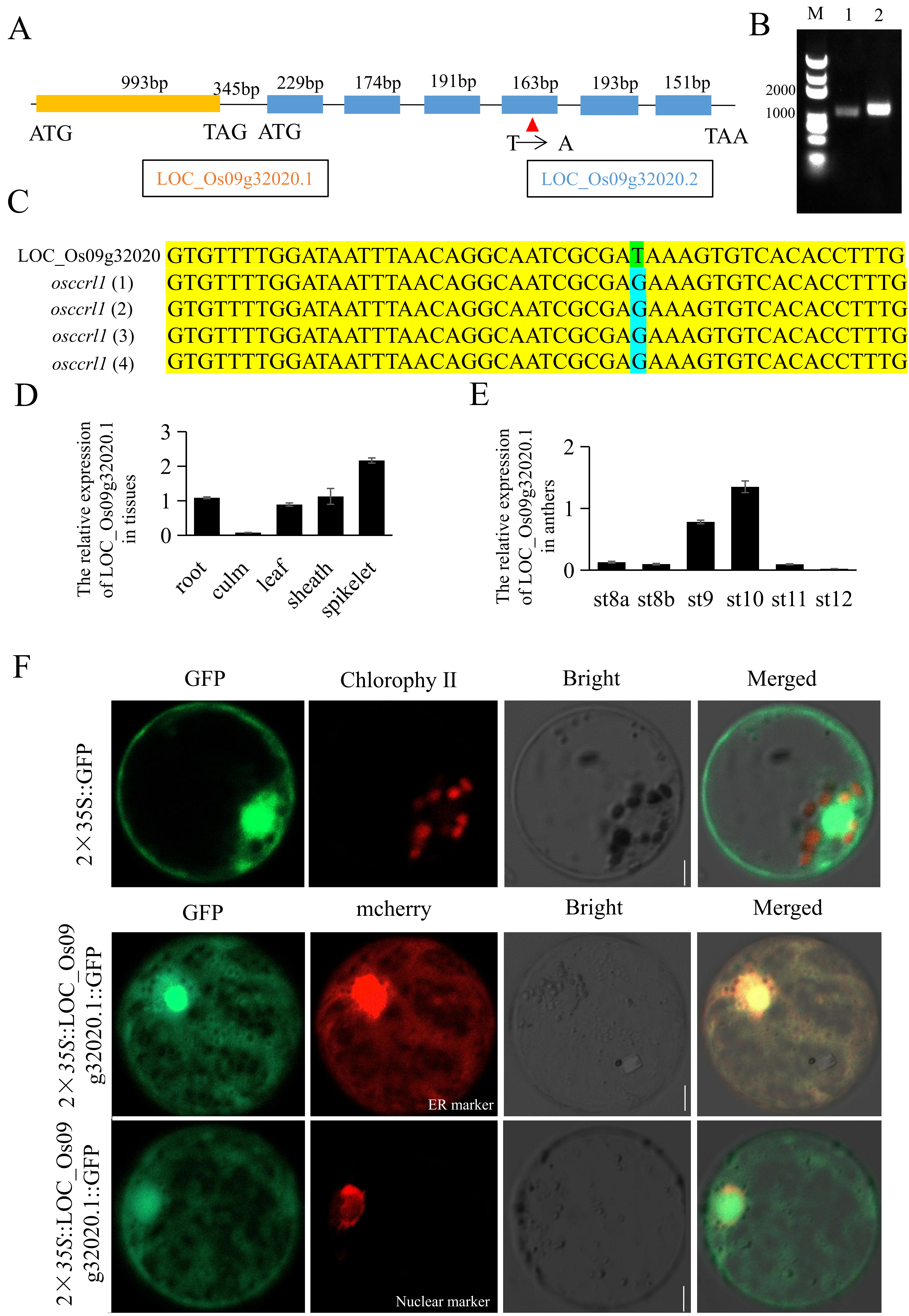


**Fig. S1.** Two *LOC_Os09g32020* transcripts, expression analysis and subcellular localization of LOC_Os09g32020.1.

**A** Schematic diagram of the two LOC_Os09g32020 transcripts. Boxes: exons; Lines: introns; Numbers: the bases number of corresponding exon or intron.

**B** Two amplified *LOC_Os09g32020* transcripts. ‘1’ means *LOC_Os09g32020.1*. ‘2’ means *LOC_Os09g32020.2.*

**C** The base difference of LOC_Os09g32020.2 genome between the sequence given online and sequenced from wild-type DNA.

**D**, **E** Abundance of *LOC_Os09g32020.1* transcripts in wild-type different tissues and anthers at different developmental stages. Data are means ± SD (*n* = 3).

**F** Subcellular localization of 2×*35S*::GFP and 2×*35S*::LOC_Os09g32020.1*-*GFP in rice protoplasts. OsHDEL-mCherry was used to indicate the endoplasmic reticulum. OsH2B-mCherry was used to indicate the nucleus. Scale bars, 5 μm.


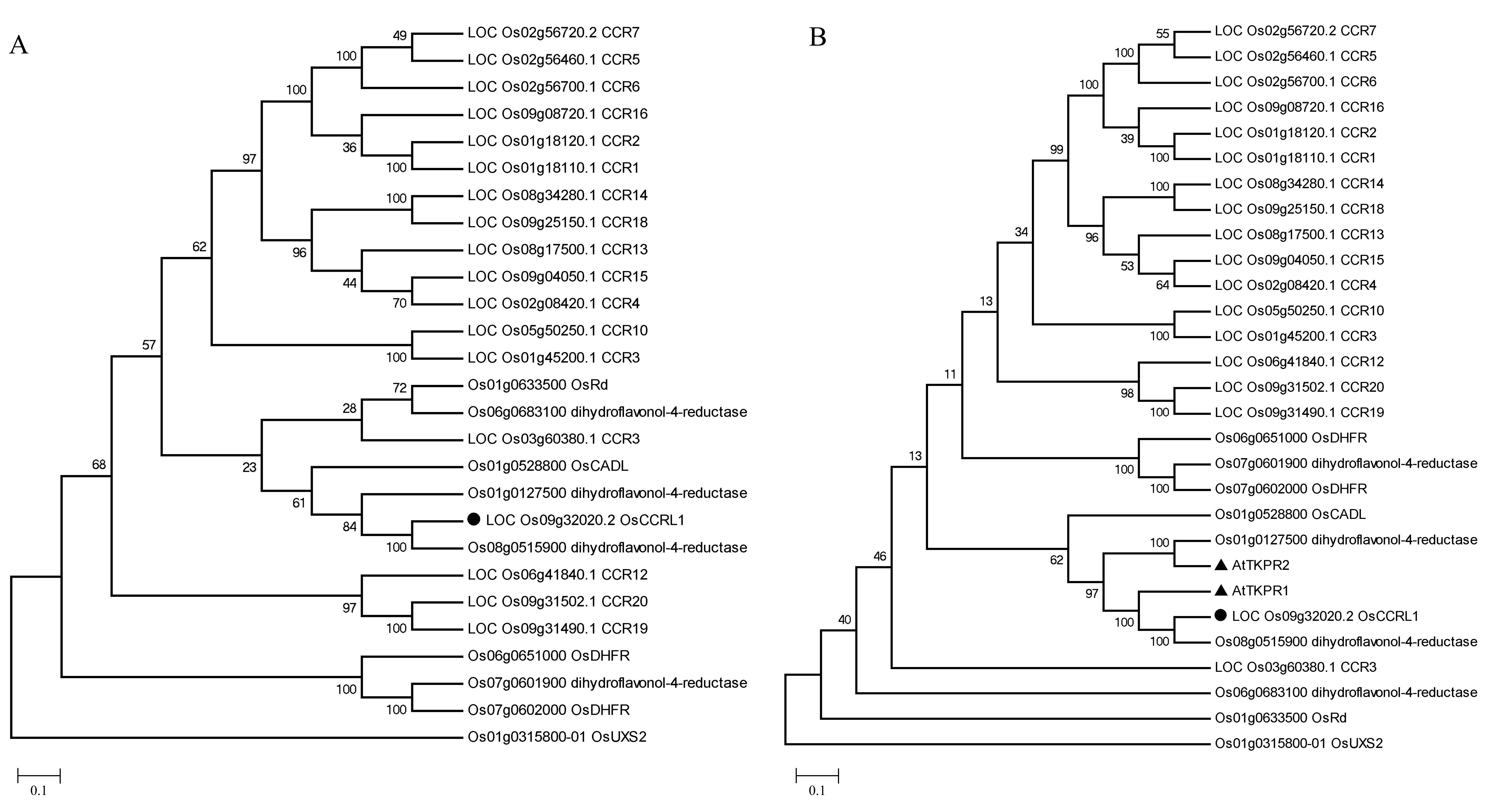


**Fig. S2.** Phylogenetic tree of OsCCRL1.

**A** An evolutionary tree of OsCCRL1 mainly based on rice. The tree was constructed using the maximum-likelihood method. Bootstrap values calculated from 1000 replicates are given at the branch nodes. The scale bar indicates the estimated number of amino acid substitutions per site.

**B** An evolutionary tree of OsCCRL1 mainly based on rice and two *Arabidopsis* TKPR. The tree was constructed using the maximum-likelihood method. Bootstrap values calculated from 1000 replicates are given at the branch nodes. The scale bar indicates the estimated number of amino acid substitutions per site.


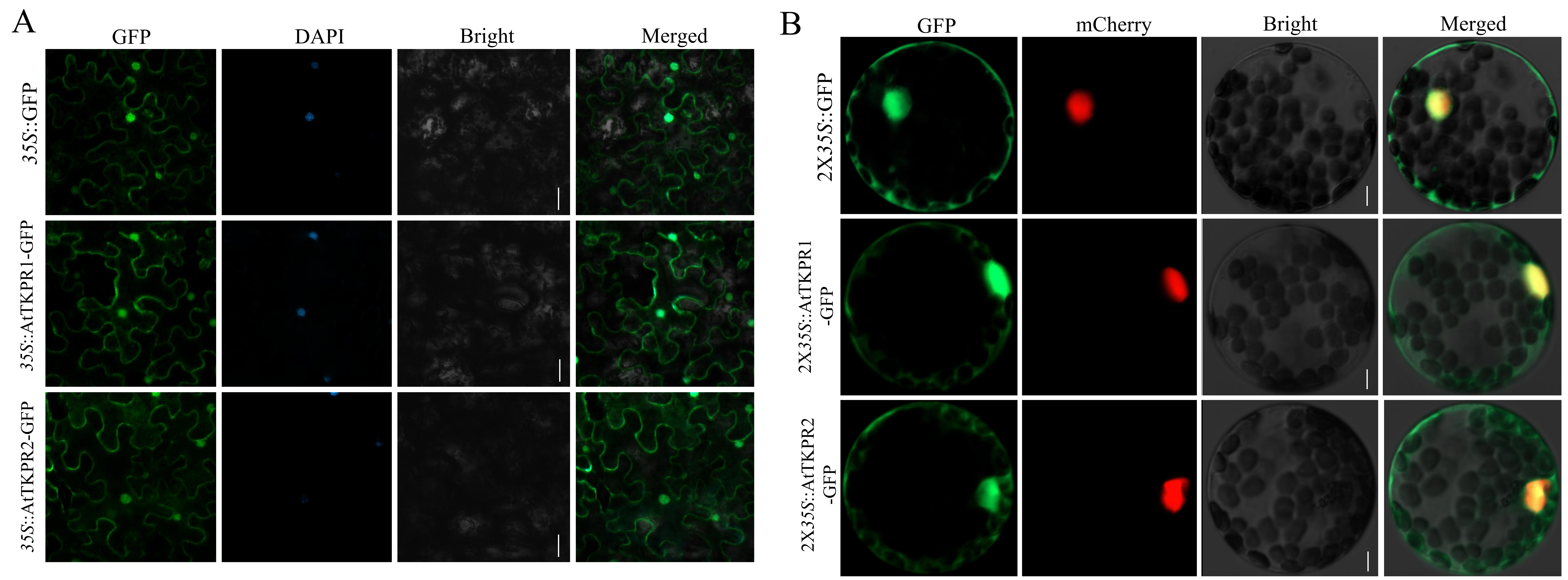


**Fig. S3.** Subcellular localization of two *Arabidopsis* TKPRs.

**A** Subcellular localization of *35S*::GFP, *35S*::AtTKPR1-GFP and *35S*::AtTKPR2-GFP with DAPI in *Nicotiana benthamiana.* Scale bar: 5 μm.

**B** Subcellular localization of 2×*35S*::GFP, 2×*35S*::AtTKPR1-GFP and 2×*35S*::AtTKPR2-GFP in *Arabidosis* protoplasts. AtVirD2NLS-mCherry was used to indicate the nucleus. Scale bars, 5 μm.


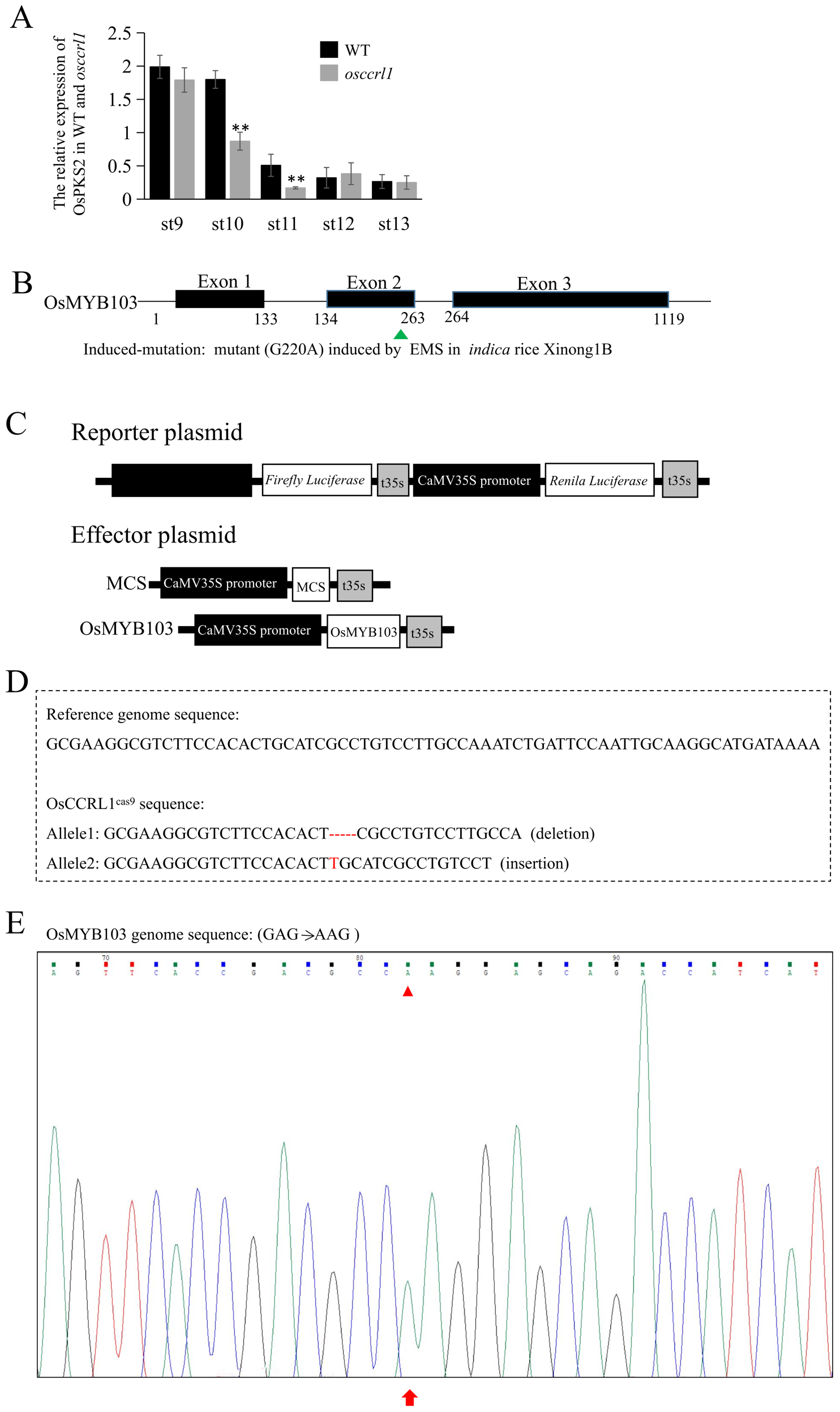


**Fig.S4.** The relative expression of OsPKS2, the schematic diagrams of transient expression assay and the sequencing of *osmyb103 osccrl1* double mutant.

**A** The relative expression of OsPKS2 in the wild-type and *osccrl1* anthers. Data are means ± SD (*n* = 3). Asterisks indicate the significance differences determined by Student’s t-test (*, 0.01≤P< 0.05; **,P<0.01).

**B** The information of OsMYB103 mutation.

**C** Schematic diagrams of the reporter and effector used in the trans-activation of the LUC reporter gene in rice protoplasts. The reporter plasmid contained the OsCCRL1 promoter sequence (1.7 kb) fused to the fifireflfly luciferase gene (LUC). OsMYB103 gene was linked to effector plasmid under the control CaMV*35S* promoter and t35s denoted the terminators of CaMV*35S*.

**D** Reference sequence and sequencing of OsCCRL1 genomic DNA in *osmyb103 osccrl1* double mutants.

**E** Sequencing of OsMYB103 genomic DNA in *osmyb103 osccrl1* double mutants.

**Supplemental Table 1 Primer Sequences.**

| Primer Name | Sequences (5'−3') |
| --- | --- |
| *OsCCRL1* mutant check-F | TTCTTACATCCTCATCATCCACGGTA |
| *OsCCRL1* mutant check-R | TTTCAATTGACTGACCTCCTTGGG |
| *OsCCRL1* com-F | ACGAATTCGAGCTCGGTACCGATGCTATCAGGTTCGCTTCAAAAC |
| *OsCCRL1* com-R | CGACGGCCAGTGCCAAGCTTGACACCAGCCATAGTACTGTAGATCC |
| U3R1-*OsCCRL1* | CCACTGCATCGCCTGTCCTTGTGCACCAGCCGGGAATCGAAC |
| gF1-*OsCCRL1* | GCACAAGGACAGGCGATGCAGTGGTTTTAGAGCTAGAAATAGCAAGTTAAAATAAG |
| *OsCCRL1* cas9 check-F | CTTCAGAGTTCAACATCATCACTTCCAG |
| *OsCCRL1* cas9 check-R | CATCCCTGATTCTTACCGTGGATGA |
| *OsCCRL1* CDS check-F | ATGTACAACATTTCTTGGTCCTCCTCC |
| *OsCCRL1* CDS check-R | CAACGGGCACTCCAGCAAGTGT |
| *LOC_Os09g32020.1* CDS check-F | ATGCTTTCCCGGATTCTGCAT |
| *LOC_Os09g32020.1* CDS check-R | ACGTTTCAACGAGTAGCTCGTCCC |
| *OsCCRL1* RT-F | ATGCTTGTTCCTGCGATAAATGG |
| *OsCCRL1* RT-R | CCTGATTCTTACCGTGGATGATGA |
| *LOC_Os09g32020.1* RT-F | CTACAACAACAAGGAGTTCCTCATCG |
| *LOC_Os09g32020.1* RT-R | GAAATCGACCTCGCAATCCGTC |
| *OsCCRL1* ISH-F | GAAATGCTTGTTCCTGCGATAAATG |
| *OsCCRL1* ISH-R | AGATTTAGGTGACACTATAGAACTGAAGCAGTGACAGACAGTTCATGTG |
| OsCCRL1 580-F | GACAGCCCAGATCAACTAGTATGTACAACATTTCTTGGTCCTCCTCC |
| *OsCCRL1* 580-R | CCCTTGCTCACCATGGATCCCAACGGGCACTCCAGCAAGTGT |
| *OsCCRL1* 1300-F | GAACACGGGGGACGAGCTCGGTACCATGTACAACATTTCTTGGTCCTCCTCC |
| *OsCCRL1* 1300-R | CCTTGCTCACCATGTCGACTCTAGACAACGGGCACTCCAGCAAGTGT |
| *LOC_Os09g32020.1* 580-F | GACAGCCCAGATCAACTAGTATGCTTTCCCGGATTCTGCAT |
| *LOC_Os09g32020.1* 580-R | CCCTTGCTCACCATGGATCCACGTTTCAACGAGTAGCTCGTCCC |
| *AtTKPR1* 580-F | GACAGCCCAGATCAACTAGTATGGATCAAGCAAAGGGAAAAGTCTGTG |
| *AtTKPR1* 580-R | CCCTTGCTCACCATGGATCCTGGAAGAACAGTAGATAAATAGCCTTGCTCGAC |
| *AtTKPR2* 580-F | TGGACGAGCTGTACAGATCTATGGATCAAGCAAAGGGAAAAGTCTGTG |
| *AtTKPR2* 580-R | AACTGCAGCCGGGCGGCCGCttaTGGAAGAACAGTAGATAAATAGCCTTGCTCGAC |
| AtTKPR1 1300-F | GAACACGGGGGACGAGCTCGGTACCATGGATCAAGCAAAGGGAAAAGTCTGTG |
| *AtTKPR1* 1300-R | CCTTGCTCACCATGTCGACTCTAGATGGAAGAACAGTAGATAAATAGCCTTGCTCGAC |
| *AtTKPR2* 1300-F | GAACACGGGGGACGAGCTCGGTACCATGTCTGAGTATTTGGTAACTGGAGGAACAG |
| *AtTKPR2* 1300-R | CCTTGCTCACCATGTCGACTCTAGAGAGCAGACCCTTCTTCTGAAAACTGATGAT |
| OsCCRL1/osccrl1 GST-F | GATCTGGTTCCGCGTGGATCCATGTACAACATTTCTTGGTCCTCCTCC |
| OsCCRL1/osccrl1 GST-R | GTCACGATGCGGCCGCTCGAGTTACAACGGGCACTCCAGCAA |
| *Os4CL3* RT-F | GGAGAGAACCCTAACCTGTACTTCAGC |
| *Os4CL3* RT-R | GCACGGAGTTGAGCGAGTAGATGT |
| *OsCHS1* RT-F | AGGATCTATTCGTTATTGCTTGTGTATTGA |
| *OsCHS1* RT-R | CTTATGATCAGACCTTCTTACATGCCAGA |
| *OsAP25* RT-F | CTTGTGCAGTCTATGTATTCTCTTCTTT |
| *OsAP25* RT-R | GTACGTACGTAATCACCTAGATATAGCC |
| *OsAP37* RT-F | TCTCAACCAGTTCTTCTTTCCTATACC |
| *OsAP37* RT-R | AAATGGGCTAAGTAAACGAGGATG |
| *C6* RT-F | ACAAGTAGATATTGGATTATTGGCATATAC |
| *C6* RT-R | GCGCATAGCACGACAGTAGCAC |
| *OsPKS2* RT-F | GACTCTGTACCGTACCTGATCCAAATCT |
| *OsPKS2* RT-R | TCGTCTTCACCGTCGTCGTCTT |
| *OsACTIN1* RT-F | GACCCAGATCATGTTTGAGACCT |
| *OsACTIN1* RT-R | GACCCAGATCATGTTTGAGACCT |
| *OsMYB103* no-GFP F | GACAGCCCAGATCAACTAGTATGGGGCGGGTGCCGT |
| *OsMYB103* no-GFP R | AACTGCAGCCGGGCGGCCGCCTACACCATGTGATTCGTGAGCTCG |
| *OsCCRL1*-pG0800 F | TCCTGCAGCCCGGGGGATCCCGAAACTCGAATCAGATTGAAGCACTAC |
| *OsCCRL1*-pG0800 R | GTTTTTGGCGTCTTCCATGGCACCTCATTTTTGTGATCATAATTAACTGAT |
| *Ubi::OsMYB103-GFP* F | GTGTTACTTCTGCAGGATCCATGGGGCGGGTGCCGTGCTGC |
| *Ubi::OsMYB103-GFP* R | CCGTCGACCTCGAGGGTACCTTAAGATCTGTACAGCTCGTCCATG |
| Chip *OsCCRL1* P1-F | CATGGAACCTGATACTATTTGCTTG |
| Chip *OsCCRL1* P1-R | GTGATCATAATTAACTGATGGAGAAGC |
| Chip *OsCCRL1* P2-F | TTCTGGGACGAGCTACTCGTTGAAA |
| Chip *OsCCRL1* P2-R | AATTAGCAAGACAATAACTAGAATTATTAAGGGAG |
| Chip *OsCCRL1* P3-F | TAGCAACTCTACACGTCAGAAAAAGGG |
| Chip *OsCCRL1* P3-R | TTTCAACGAGTAGCTCGTCCCAGAA |
| Chip *OsCCRL1* P4-F | GCGGCTTCCGAAGATCAAGATCAA |
| Chip *OsCCRL1* P4-R | CTTGCAGCAGCAGGATTAGAAGAGA |
| Chip *OsCCRL1* P5-F | CATCATCGAGACGGATTGCGAG |
| Chip *OsCCRL1* P5-R | TTGATCTTGATCTTCGGAAGCCG |
| Chip *OsCCRL1* P6-F | TCGTCGTCAGGAGCGCCAGCCTCC |
| Chip *OsCCRL1* P6-R | GGCAGGTGAAGCTGCGCAGCGTC |
| Chip *OsCCRL1* P7-F | CAGATTGAAGCACTACCATTGTTCG |
| Chip *OsCCRL1* P7-R | CTTATGAGGGCAGTTCGTTGATCCG |
| Chip *OsCCRL1* P8-F | AGGTTCAGCAGCTTCCATGGTGTT |
| Chip *OsCCRL1* P8-R | CGAACAATGGTAGTGCTTCAATCTG |
| *OsMYB103* 28a-F | AGCAAATGGGTCGCGGATCCATGGGGCGGGTGCCGTGCTGC |
| *OsMYB103* 28a-R | GTGGTGGTGGTGGTGGTGCTCGAGCACCATGTGATTCGTGAGCTCGTCGG |
| Label P2s probe F | **Biotin**-ACGTTAGCTGCTGCTATTAGGGTTAAAAGTTTTGGAATCAATCATTTGTTTCTTACTTGGAGTTCGCCTGGTTACTCTGGCAGCTGTAAATGA |
| P2s probe R | TCATTTACAGCTGCCAGAGTAACCAGGCGAACTCCAAGTAAGAAACAAATGATTGATTCCAAAACTTTTAACCCTAATAGCAGCAGCTAACGT |
| Label P2s mutant probe F | **Biotin**-ACGTTAGCTGCTGCTATTAGATCGAAAAGTTTTGGAATCAATCATTTGTTTCTTACTTGGAGTTCGCCTATCGACTCTGGCAGCTGTAAATGA |
| P2s mutant probe R | TCATTTACAGCTGCCAGAGTCGATAGGCGAACTCCAAGTAAGAAACAAATGATTGATTCCAAAACTTTTCGATCTAATAGCAGCAGCTAACGT |
| Label P7s probe F | **Biotin**-ATCAATTTTAGATTTCTAAACCGAATCTTGTTGCGAGTTC |
| P7s probe R | GAACTCGCAACAAGATTCGGTTTAGAAATCTAAAATTGAT |
| Label P7s mutant probe F | **Biotin**-ATCAATTTTAGATTTCTACGTAGAATCTTGTTGCGAGTTC |
| P7s mutant probe R | GAACTCGCAACAAGATTCTACGTAGAAATCTAAAATTGAT |

**Supplemental Table 2** Peptide Sequences for phylogenetic tree.

| ID | Peptides Sequences (N−C) |
| --- | --- |
| LOC_Os08g34280.1 CCR14 | MTVIDGAVAADAGGAAAAVVQPGNGQTVCVTGAAGYIASWLVKLLLEKGYTVKGTVRNPDDPKNAHLKALDGAGERLVLCKADLLDYDAICRAVAGCHGVFHTASPVTDDPEQMVEPAVRGTEYVINAAAEAGTVRRVVFTSSIGAVTMDPNRGPDVVVDESCWSDLDYCKETRNWYCYGKAVAEQAAWEAARRRGVELVVVNPVLVIGPLLQPTVNASVAHILKYLDGSASKFANAVQAYVDVRDVAAAHLLVFESPSAAGRFLCAESVLHREGVVRILAKLFPEYPVPTRCSDEKNPRKQPYKMSNQKLRDLGLEFRPASQSLYETVKCLQEKGHLPVLAAEKTEEEAGEVQGGIAIRA* |
| LOC_Os09g25150.1 CCR18 | MTVVVVADDAAAAAAAAQQQEELPPGHGQTVCVTGAAGYIASWLVKLLLERGYTVKGTVRNPDDPKNAHLKALDGADERLVLCKADLLDYDSIRAAVDGCHGVFHTASPVTDDPEQMVEPAVRGTEYVIKAAAEAGTVRRVVFTSSIGAVTMDPNRGPDVVVDESCWSDLEFCKKTKNWYCYGKAVAEQEACKAAEERGVDLVVVSPVLVVGPLLQPTVNASAVHILKYLDGSAKKYANAVQAYVDVRDVAAAHVRVFEAPEASGRHLCAERVLHREDVVHILGKLFPEYPVPTRCSDEVNPRKQPYKMSNKKLQDLGLHFIPVSDSLYETVKSLQEKGHLPVLSKEIPEELNGVPA* |
| LOC_Os02g56720.2 CCR7 | MSSISNDNNGDQKRQQQQQLVCVTGAGGFIGSWVVRELLLRGYRVRATVRDPADRKNAHLLALEGAHERLSLRRADVLDFAGLLAAFAGCHGVFHVACPLSNRDPELMAVAVDGTRNVMNAAADMGVRRVVFTSSYGAVHMNPNRSPDAVLDESCWSDPEFCRQKDMYCYAKTMAEMAATEEAAKRGLELAVVVPSMTMGPMLQRALNLSSTHVANYLTGAKKSYPNAVAAYVDVRDVARAHALVYERHDARGRYLCIGAVLHRAQLLQMLMDLFPQYTIASKCDDKGKPMVKPYEFSNQRLKDLGLEFTPLRKSLYDAVMCMQRNGHLPVVLP* |
| LOC_Os02g56700.1 CCR6 | MSSNNSMEANNGDDEKKQEQVVCVTGAGGFIGSWVVKELLLRGYRVRGTARDPRKNAHLLDLEGAKERLTLCRADVLDFASLRAAFAGCHGVFHIASPVSKDPNLVPVAIEGTRNVMKAAADMGVRRVVFTSSYGAVHMNPNRSPDAVLDESCWSDPEFCQREDIYCYAKMMAEKTATEEASRRRLQLAVVVPCVTVGPILQPSVNFSCHHVVRYLTGAAATYPNAVAAYADVRDVARAHVLVYEHHGARGRYLCIGTVIHRAELLRMLKELFPQYPVTSKCEDEGNQMVKPYKFSNQRLRDLGLEFTPLRKSLHEAIECLQRKGHLPVVTVAQQRACL* |
| LOC_Os02g56460.1 CCR5 | MSSNFEANNNNNGEKQLVCVTGAGGFIGSWVVKELLIRGYHVRGTARDPADSKNAHLLELEGADERLSLCRADVLDAASLRAAFSGCHGVFHVASPVSNDPDLVPVAVEGTRNVINAAADMGVRRVVFTSSYGAVHMNPNRSPDAVLDETCWSDYEFCKQTDNLYCCAKMMAEMTATEEAAKRGLELAVVVPSMTMGPMLQQTLNFSTNHVARYLMGTKKSYPNAVAAYVDVRDVARAHVLVYERPEARGRYLCIGTVLHRAELLRMLRELFPRYPATAKCEDDGKPMAKPYKFSNQRLKDLGLEFTPLRKSLNEAVLCMQQKGHLPLIYPVPKRAYL* |
| LOC_Os01g18120.1 CCR2 | MVTGRSEQMVCVTGAGGFIGSWLVKELLHRGYFVRGAMREPADIKNAHLHVLDGAREGLSLYRADVLDRNSLRAAFALCDGVFHVASPVSNDPELLPAAIEGTKNVINAAADMGVKRVVFTSSYGAVHMNPNRRSDQIVDESCWSDLEFCKQTQNWYCYAKMLAERTAMEEASKRGVNLLVVVPAVTVGEMLQPTLNASVHRVATYMRGTKSAYPNAVAAYVDVRDVARAHALVYEHPDARGRYLCIGSVLHRSEFVRLLRELFPQYPITSRCKDNSKPMVKPYKFSVQRLETLGMQFTPLKESLYRTVISLQDKGHLPAAISRRSAL* |
| LOC_Os01g18110.1 CCR1 | MVTGVEQMVCVTGAGGFIGSWLVKELLHRGYAVRAAVRDPEGRKNAHLHALERAKRRLSLHRADVLDCNSLRAAFNLCDGVFHVASPVSDDPELLPTAIEGTKNVINAAADMGIKRVVFTSSYGAAHMNPNRRSDQTLDETCWSDLEFCKQTQNWYCYAKTVAEKTATEEASKRGVQLLVVVPAVTVGEMLQPTLNASVYRVATYMRGTKSAYPNAVAAYVDVRDVARAHALVYEHPDARGRYLCIGSVLHRSEFVRLLRELFPQYPITTRCEDNSKPMVKPYQFSVQRLEALGMQFTPLKESLYKTVISLQDKGHLPAISPRSAL* |
| LOC_Os09g08720.1 CCR16 | MMAKAEGGKMVCVTGAGGFIGSWVVKELLLRGYAVRGTARDPSSQKNSHLQKLEGAKERLCLNYADVMDYDSLSVAFNGCEGVFHVASPVSVDPRLVPVAVEGTKNVINAAADMGVRRVVFTSTFGAVHMDPNRSHDTVVDESCWSNLEFCKQKDWYCYAKTVAEMVAAEQASKRGIQLVVVLPAMTLGQMLQSTINPSIRHIADFLNGSRKTHRNAVAGYVDARDVARAHALVYEDPKAHGRYLCIASVLHRSELIQMIRELFPQYPITCNKCEDSKQMVQPFKFSNQRLRDLGLTFTPIKESLYNTLICLREKGHLPPYSSL* |
| LOC_Os03g60380.1 CCR3 | MASGGGGGGEGETVLVTGASGFIGSCLVRRLLARGYSVHAAVLNPDDKAETDHLHALAAAGGGEGRRLRVFPGDLLDGAALLAAARGCSGVFHLASPCIVDRVLDPQAQLMVPAVEGTLNVLRAAKDAGGVRRVVVTSSISAIVPSPGWPAGEVRDERCWTDLDYCEKNGVWYPASKTLAEKAAWKFAEENGLDVVVVNPGTVMGLVIPPTINASMAMLVRLLEGCTEEYADFYMGPVHVEDVALAHILLYENPSASGRHLCVQSIAHWSDFASKVAELYPEYKVPKLPKETQPGLVRAEAASKKLIALGLQFSPMEKIIRDSVESLKSRGFIS* |
| LOC_Os06g41840.1 CCR12 | MLGGDGRTKTVCVTGAGGFVASWLVKLLLSRGCYTVHGTVRDPGDAKNAHLMSLDGAAERLRLFKADLLDYGSVAAAIAGCDDVFHVACPVLLSAPNPEVDILAPAVTGTTNVLKACSEAKVGRVVVVSSVSAAMVNPNWSEGKAIDEDCWSDVDYCRATKNWYTLGKTLAEIEAFDYAKRSGLDLVTLCPSLVIGPLLQPTVNASSTVILGCLKGDCEVKIKLRNFVDVRDVADALLLLYETPGVSGRYICSSHARRMPHIIDLLKSWYPGYKFADKFVEVSDEPQFNSGKLEKLGWKIKPFEETLRDSVESYRAAGVLD* |
| LOC_Os09g31502.1 CCR20 | MAAMASPPPPTRVCVTGAGGFIGSWLVKLLLSRGYAVHATLRDPCDPKNAHLKQLDGASEMLSLFKADVLDAGELSAAIAGCEGVFHVASPVPGDKIVDPELEVMAPAVKGTLNVLEVCSSSKKVQKVVVVSSTAAVHYNPNWPPGKPKDESCWSDRKICMEKKEWYSASKVIAEKMALEYAEKKGLNVVTVCPCLVFGPQLQPTVNTSNELLIYITKGGPNVMRNMLLHIVDVRDVAEALILVYEKPESSGRYLCAPYHISPKATVEFLKNIYPNYNYVKCSAEVNGKTEIFTPISSEKLKSLGWKPRKLEETLTDSIEYYEKTGILQDAGGRPCVLPYLFHFLVEN* |
| LOC_Os09g31490.1 CCR19 | MTPPPPPPRRPVCVTGAGGFTGSWLVKLLLSRGYAVHATLRDPDDPKNAFLKQLENAPENLRLFKADVLDGGSLTAAFAGCEGVFHPATPVPEHKTVDPEKEMLAPAVKGTRNVLEACSAASVQKLVVVSSICAVCFNPSLPRDRLIDETCWSDKKSCKENENWYCLAKTEAEEMALEYSEKNGLHVITVCPGVIFGPLLQTVLLNTSSKVLLYIMKGGPDALSNKFFPIVDVRDVADALLLVYDKAGPSERYICSQEQMDMRDLLDLMKSMYPNYSYTAKVVDVDMTTSVELTSEKLKKLGWKPRKLEETLVDSVESYKKAGFVDDEPCRLPHLYRAPDAQE* |
| LOC_Os05g50250.1 CCR10 | MGVLRSTQSMEAEVEEMRAALALAPLGRHGAWRSGAAAKREAGAEEGAAPEARTVCVTGGISFVGLAVVDRLLRHGYAVRLALETQEDLDKLREMEMFGENGRDGVWTVMANVMDPESLNQAFNGCVGVFHTSSLIDPGGISGYTKHMAILEARAAEQVVEACVRTESVRKCVFTSSLLACVWRQSYPHHRRRFPAIIDESCWSDESFCRDNKLWFALGKTMAEKAAWRAARGRDLKLVTICPALVTGPGFRRRNSTPSIAYLKGAHAMLAEGLLATADVERVAEAHVRVYEAMSGGGAAGGRYICYDHVVRRGEEFAELQRQLGLPITGVAAASRPGYSDDGDVGGDGRFALCNGKLARLVSSRRRCTYDVYYPASYD* |
| LOC_Os01g45200.1 CCR3 | MRAALLHGHGGGAAAAAAAGWRPSAGDADVKRTAGGDGGAAGPRTVCVTGGISFVGFAVVDRLLRHGYTVRLALETQEDLDKLREMEMFGEDGRDGVWTVMANVTDPESLHRAFDGCAGV  FHTSAFVDPGGMSGYTKHMASLEAKAAEQVIEACVRTESVRKCVFTSSLLACVWRQNYPHDRRFPTIIDENCWSDESFCRDNKLWFALGKTAAEKTAWRAARGRDLKLVTVCPALVTGPGFRRRNSTASIAYLKGARAMLADGLLATASVETVAEAHVRVYEAMGDNTAGGRYICYDHVVKRPEEFAELERQLGIPRRAAAAAAAQDSGDRPARFDLCRQKLARLMSTRRRCTYDDYYSVAFD* |
| LOC_Os08g17500.1 CCR13 | MGVEKTTANGGSGAAAVSGGGRTVCVTGAGGFIASWLVKLLLEKGYAVRGTVRNPDDAAKNAHLMALAGAAERLTLVRAELLDKESLAAAFAGCEGVFHTASPITDDPEKMIEPAVSGARNVITAAADAGGVRRVVMTSSIGAVYMGGGGGEEVDETCWSDLDHCRDTGNWYCYAKTVAEQAAWELAKERRLDLVVVNPSLVLGPLLQRGVNASTWHVLKYLDGSARTYADAAQAYVHVRDVADAHARAYESPAARGRYLCAGRTLHRGEVCRILAALFPGYPVPTRCKGDAGETAEGCRFSSRKLAELGVAVMPASQCLYDTVVSLQDKGLLPFVPAAAMP* |
| LOC_Os09g04050.1 CCR15 | MPTDETAAAAPATTALSGHGCTVCVTGAGGFIASWLVKRLLEKGYTVRGTVRNPMDPKNDHLRALDGAGERLVLLRADLLDPDSLVAAFTGCEGVFHAASPVTDDPEKMIEPAIRGTRYVITAAADTGIKRVVFTSSIGTVYMNPYRDPNKPVDDTCWSDLEYCKRTENWYCYAKTVAEQGAWEVARRRGVDLVVVNPVLVLGPLLQATVNASTEHVMKYLTGSAKTYVNAAQAYVHVRDVAEAHVRVYDCGGARGRYICAESTLHRGDLCRALAKLFPEYPVPSRCKDEAAPPVKGYLFSNQRLRDLGMDFVPVRQCLYETVRSLQDKGLLPVLPPTADDHHHPSS* |
| LOC_Os02g08420.1 CCR4 | MAAAVVCVTGAGGFIGSWIVKLLLARGYAVRGTSRRADDPKNAHLWALDGAAERLTMVSVDLLDRGSLRAAFAGCHGVIHTASPMHDDPEEIIEPVITGTLNVVEVAADAGVRRVVLSSTIGTMYMDPRRDPDSPLDDSFWSDLDYCKNTKNWYCYAKTIAERKAWEVARGRGVDMAVVIPVVVLGELLQPGMNTSTKHILKYLTGEAKTYVNESHAYVHVVDAAEAHVRVLEAPGAGGRRYICAERTLHRGELCRILAGLFPEYPIPTRCRDEINPPKKGYKFTNQPLKDLGIKFTPVHEYLYEAVKSLEDKGFIKKTSNTKELHRQSSPPQNSPASMLMSKL* |
| LOC_Os09g32020.2 OsCCRL1 | MYNISWSSSSRIFLCCAYMSRLICFRVQHHHFQFEKMVISSKGKVCVTGASGFVASWLIKRLLEAGYHVIGTVRDPSNREKVSHLWRLPSAKERLQLVRADLMEEGSDFDAVMACEGVFHTASPVLAKSDSNCKEEMLVPAINGTLNVLKSCKKNPFLKRVVLTSSSSTVIRDESKHPEISLDETIWSSVALCEKLQLWYALAKISAEKAAWEFAKENNIDLVTVLPSFVIGPSLSHELSVTASDILGLQGDTDRFISYGRMGYVHIDDVASCHILVYEAPQATGRYCNSVVLDNNELVALLAKQFPIFPOPRSLRNPYKQSYELNSTKIQQLGFKFKGVQEMFGDCVESLKDQGHLLECPL* |
| Os08g0515900 dihydroflavonol-4-reductase | MENTTKGKVCVTGASGYVASWLVKRLLESGYHVLGTVRDPGNHKKVGHLWNLTGAKERLELVRADLLEEGSFDDAVMACEGVFHTASPVITETDSSKAAVLDSAINGTLNVLRSCKKNPSLKRVVLTSSSSTVRLKDEADLPPNVLLDETSWSSMEFCESLQIWYAIAKTLAEKAAWEFAKENGIDLVAVLPTFVVGPNLSHELSPTTTDVLGLFQGETTKFTMYGRMGYVHIDDVASCHILLYETPRAAGRYICNSAVLDVNELVTLLARRFPSYPIPKRSRTPAKWNFFFSAD* |
| Os01g0633500 OsRd | MDFESEDPENEVVKPTVEGMLSIMRACRDAGTVKRIVFTSSAGTVNIEERQRPSYDHDDWSDIDFCRRVKMTGWMYFVSKSLAEKAAMEYAREHGLDLISVIPTLVVGPFISNGMPPSHVTALALLTGNEAHYSILKQVQFVHLDDLCDAEIFLFESPEARGRYVCSSHDATIHGLATMLADMFPEYDVPRSFPGIDADHLQPVHFSSWKLLAHGFRFRYTLEDMFEAAVRTCREKGLLPPLPPPPTTAVAGGDGSAGVAGEKEPILGRGTGTAVGAETEALVK* |
| Os01g0528800 OsCADL | MSSESEAAPGTGKLVCVTGASGYIASWLVRLLLARGYTVRATIRDTSDPKKTLHLRALDGANERLHLFEANLLEEGSFDAAVNGCDCVFHTASPFYHNVKDPKAELLDPAVKGTLNVLGSCKKASIRRVIVTSSMAAVAYNGKPRTPDVVVDETWFSVPEICEKHQQWYVLSKTLAEEAAWKFSKDNGFEIVTVNPAMVIGPLLQPSLNTSAEAILKLINGSSSTYPNFSFGWINVKDVALAHILAYEVPSANGRYCMVERVAHYSELVQIIREMYPNIPLPDKCADDKPSVPIYQVSKEKIKSLGLELTPLHTSIKETIESLKEKGFVTFDSSNL* |
| Os01g0127500 dihydroflavonol-4-reductase | MPEYCVTGGTGFIASHLIRALLAASHTVRATVRDPEDEAKVGFLWELDGASERLQLVKADLMVEGSFDDAVRGVDGVFHAASPVVVVGNSSSNNGKPNDDDDEEEVQQRLVEPIVRGASNVLRSCARASPRPRRVVFTSSCSCVRYGAGAAAALNESHWSDAAYCAAHGLWYAYAKTLAEREAWRLAKERGLDMVAVNPSFVVGPILSQAPTSTALIVLALLRGELPRYPNTTVGFVHVDDAVLAHVVAMEDARASGRLICSCHVAHWSEIVGSLRERYPGYPIPAECGSHKGDDRAHKMDTAKIRALGFPPFLSVQQMFDDCIKSFQDKGLLPPHA* |
| Os06g0683100 dihydroflavonol-4-reductase | MGSIGGGGSPEERAAAGGPVCVTGSTGYVGSWLVRALLRRGYRVHATARDPDKAWRVFSAVEEGKDQLRVFRADMAGEGSFDAAATGCVAFFHVAASMDIHVPPQNGNDNNIEEHVRTRVLEPATRGTINVLQSCVRAGTVRRVVFTSSISTMTAATTTAATGRRKAVVDESCLRAAADVWNTKPIGWVYILSKLMTEEAAFGFARENGINLASLVLPTVAGPFLTPNVPTSIQLLLSPITGDPKLYSLLASVHSRFGCVPLAHIQDVCDAHVFLMETEQADGRYLCAGGSYPMAQIAQILSLHYPPFKPAKRLSKDFHGSNPSVVSSKRLRDLGFRFEYDVEEIIKNSVVQCVDHGFLQDPDSSNC* |
| Os06g0651000 OsDHFR | MAEEGRSGGVAGDGVRVCVTGGAGFIASWLVKKLLERGCIVHATLRSMGDEEKAGLLRRLVGAAERLRLFEADLFDAATFAPAIAGCQFVFLIATPYGLEASNSKYKNTADAAVDAVREILRQCAESKTVKRVIHTASISTASPLIDVPGAGVGAAGYRDFIDESCWTPLDVDYPLRSAHFDVISPPLFFLLQSRTR* |
| Os01g0315800 OsUXS2 | MASELTYRGGAGAPGSASDGGEYSPKPSKPLSWLTRAARYAAAEHRPAFALAGMLLAAALFSLYAPSSDASSSAATTTTTTFSHLSSLPSSSAASLHESAGGKVPLGLRRRALRVLVTGGAGFVGSHLVDRLVERGDSVIVVDNFFTGRKDNVAHHLANPRFEVIRHDVVEPILLEVDQIYHLACPASPVHYKYNPIKTIKTNVVGTLNMLGLAKRIGAKFLLTSTSEVYGDPLQHPQVETYWGNVNPIGVRSCYDEGKRTAETLTMDYHRGANLEVRIARIFNTYGPRMCIDDGRVVSNFVAQALRKEPLTVYGDGKQTRSFQYVSDLVEGLMSLMEGEHIGPFNLGNPGEFTMLELAKVVQDTIDPNARIEFRPNTADDPHKRKPDITRAKELLGWEPKVPLREGLPLMVTDFRKRIFGDQEA* |
| Os07g0601900 dihydroflavonol-4-reductase | MTSSSSSRVCVTGASGYIATCLIKKLLQRGCVVHATLRNLGDEKKTAPLRELPGAAERLVLFEADMYDADTFEPAIAGCEFVFLLATPLQHDPRSTKYKNTTEAAVDAMRIILQQCERSKTVRRVIHTASVTAASPLREDGGEGYKDFINESCWTPLDHSHSYNNTMVDVLSPFHSVSHFSISHIYVNVNKSRHIYININVKNTRMTYIVKQRKYVINQLRISC* |
| Os07g0602000 OsDHFR | MSSSRPVCVTGGSGYIATCLIKKLLQRGCGVHATLRNLGDEKKTALLRRMPGAAERLVLFEADMYDAATFEPAIAGCEFVFLLATPLIHDPLSTKYKNTTEAAVDAMHIILQQCERSKTVRRVIHTASVTAASPLREDGEGYKDFINESCWTPLDLSNRYSNVMMDAYVSSKTLTEKLLLRYNESESRAFEVVTLTCALIGGDTDTTQLYHSLSIPLIVSPLTGQELYHGGLKSLQALLGSVPLAHIDDICEAHIFCMEQQPSIAGRFLCAVGYPNMQDYVDRFAVKYPEIAIKLQGVIGKDVRVKADTNKLGDLGFKYKFTVEETLDSSVECAKRLGLL* |
| AtTKPR1 | MDQAKGKVCVTGASGFLASWLVKRLLLEGYEVIGTVRDPGNEKKLAHLWKLEGAKERLRLVKADLMEEGSFDNAIMGCQGVFHTASPVLKPTSNPEEEILRPAIEGTLNVLRSCRKNPSLKRVVLTSSSSTVRIRDDFDPKIPLDESIWTSVELCKRFQVWYALSKTLAEQAAWKFSEENGIDLVTVLPSFLVGPSLPPDLCSTASDVLGLLKGETEKFQWHGQMGYVHIDDVARTHIVVFEHEAAQGRYICSSVISLEELVSFLSARYPSLPIPKRFEKLNRLHYDFDTSKIQSLGLKFKSLEEMFDDCIASLVEQGYLSTVLP* |
| AtTKPR2 | MSEYLVTGGTGFIASYIIKSLLELGHTVRTTVRNPRDEEKVGFLWEFQGAKQRLKILQADLTVEGSFDEAVNGVDGVFHTASPVLVPQDHNIQETLVDPIIKGTTNVMSSCAKSKATLKRIVLTSSCSSIRYRFDATEASPLNESHWSDPEYCKRFNLWYGYAKTLGEREAWRIAEEKGLDLVVVNPSFVVGPLLGPKPTSTLLMILAIAKGLAGEYPNFTVGFVHIDDVVAAHVLAMEEPKASGRIICSSSVAHWSEIIELMRNKYPNYPFENKCSNKEGDNSPHSMDTRKIHELGFGSFKSLPEMFDDCIISFQKKGLL* |
